# A video-game-based method to induce states of high and low flow

**DOI:** 10.3758/s13428-023-02251-w

**Published:** 2023-10-20

**Authors:** Freya Joessel, Swann Pichon, Daphne Bavelier

**Affiliations:** 1 Faculté de Psychologie et Sciences de L’Education, (FPSE), Université de Genève, Boulevard du Pont d’Arve, 40, 1205 Geneva, Switzerland; 2 Campus Biotech, Chemin des Mines, 9, 1202 Geneva, Switzerland; 3 Geneva School of Health Sciences, HES-SO University of Applied Sciences and Arts Western Switzerland, Geneva, Switzerland

**Keywords:** Psychological flow, Video game, Psychophysiology, Intrinsic motivation, Effort

## Abstract

Flow has been defined as a state of full immersion that may emerge when the skills of a person match the challenge of an activity. It is a special case of being on task, as during flow, keeping focused on the task feels effortless. Most experimental investigations of the neural or physiological correlates of flow contrast conditions with different levels of challenge. Yet comparing different levels of challenge that are too distant may trigger states where the participant is off task, such as boredom or frustration. Thus, it remains unclear whether previously observed differences ascribed to flow may rather reflect differences in how much participants were on task—trying their best—across the contrasted conditions. To remedy this, we introduce a method to manipulate flow by contrasting two video game play conditions at personalized levels of difficulty calibrated such that participants similarly tried their best in both conditions. Across three experiments (> 90 participants), higher flow was robustly reported in our high-flow than in our low-flow condition (mean effect size *d* = 1.31). Cardiac, respiratory, and skin conductance measures confirmed the known difference between a period of rest and the two on-task conditions of high and low flow, but failed to distinguish between these latter two. In light of the conflicting findings regarding the physiological correlates of flow, we discuss the importance of ensuring a low-flow baseline condition that maintains participants on task, and propose that the present method provides a methodological advance toward that goal.

## Introduction

Flow is a state of intense attentional focus that has been defined to describe an optimal experience, enjoyable and intrinsically motivating, which can emerge when the challenge of an activity is matched to the skills of the person (Csikszentmihalyi, 1974; Nakamura & Csikszentmihalyi, 2014). Importantly, flow is a special case of being on task while remaining focused on that task feels effortless (Csikszentmihalyi, 1990; Ullén et al., 2010) and contrasts with other on-task behaviors, not associated with flow, which typically require an effortful maintenance of attention. Identifying unobtrusively whether or not someone is in flow state is an important and yet-unresolved issue in experimental works aiming at investigating the psychological and physiological correlates of flow. Indeed, like sleep, one cannot get in and out of flow on demand, and asking someone about their flow level will take them out of that state. Experimental investigations of flow usually contrast conditions of low, medium, and high challenge in the same task, where the medium condition is assumed to be the “flow” condition, as it is where the challenge of the task is aimed at being the closest to the skills of the participant. It is thus crucial to choose a task and difficulty levels such that not only can high flow emerge in one condition, but the participant also remains on task in the other condition(s) that serve(s) as the baseline state. Indeed, as the difficulty of the task becomes either low or high, mental states such as boredom (low difficulty) or frustration (high difficulty) may emerge, thus pushing the participant off the task. It would then be unclear whether differences between the flow condition and such baseline conditions may be ascribed to flow state rather than differences in being on or off task. We review below the various video game-based paradigms that have been used to experimentally manipulate flow, before introducing a methodical approach that not only ensures different flow levels, but also ensures that participants remain on task, truly committed to trying their best in conditions of different challenge levels (replacing studies like those of Bian et al., 2016, that have only one flow condition).

Many real-life activities have been associated with the experience of flow, but few can be experimentally manipulated in a lab. Video game play behaviors offer this possibility and are known to elicit strong flow experiences (Csikszentmihalyi, 2014; Sweetser & Wyeth, 2005). Some have even suggested that making boring tasks playful is a way to induce flow and overcome boredom (Mathwick & Rigdon, 2004). Therefore, a majority of experimental studies in the literature have resorted to some sort of play behavior to induce flow, with a strong historical focus on sports playing. Yet, to extract meaningful neural or physiological markers of flow, sports manipulations are far from optimal given the large muscle activity and motion they entail. As such, studies that investigated the neural and physiological correlates of flow have often used computerized tasks, especially video games, in which they manipulated the level of difficulty across different experimental conditions.

Using a race game, Tozman et al. (2015) contrasted three conditions where difficulty was varied with the goal of inducing different levels of flow: an easy condition where participants drove on a straight road, a medium-difficulty condition where the racetrack was slightly bendy, and a hard condition where participants played on a very bendy road against nine opponents stronger than them. The same principle was used in Harris et al. (2017), who asked participants to complete, in a race game, ten laps on tracks at three fixed levels of difficulty. In a somewhat similar paradigm, Peifer et al. (2015) had participants play Pac-Man through five increasing levels of difficulty, ranging from very easy to very hard, with the third level being of medium difficulty. The levels of difficulty were determined first by asking participants to rate the difficulty of ten levels, five of which were then selected for the final experiment. Unlike other studies, Nacke and Lindley (2010) did not use a high-difficulty condition. Using the game Half-Life 2 as a basis, they implemented a first, easy condition where the level was designed to be very linear and repetitive, with no progression in difficulty, a second flow condition designed according to criteria for a flow experience in terms of pace and difficulty progression, and a third condition designed to be more narratively rich, with high potential exploration but with less emphasis on fitting the pace to the player.

In all these studies, the medium level of activity was framed as the flow condition of interest. However, the difficulty in the “medium” condition was not fixed for all and not titrated to the individual skills of each participant. As such, these paradigms contrasted fixed difficulty levels in rather similar ways as 1990s literature investigating the physiological correlates of effort (Althaus et al., 1998; Backs & Seljos, 1994). Although this allows us to understand how flow, task difficulty, and effort relate to each other, it is generally agreed in the literature that the flow state cannot be reduced simply to different levels of task difficulty or effort. Rather, flow corresponds to a unique psychological state that may emerge when the challenge of the task meets the skill level of the individual, which to be accurately investigated requires a baseline condition that also keeps participants on task (instead of considering baseline low-flow conditions, where participants are likely to become bored or too frustrated and be off task).

More recent studies have attempted to tailor the difficulty of a “medium” flow condition to the skills of participants to ensure it was associated with an optimal level of challenge. We consider below two approaches to doing so. A first approach is to use an adaptive algorithm that adapts task difficulty in real time while participants are playing. For example, in Keller et al. (2011), participants played the game Tetris in one of three individually tailored conditions of difficulty: easy, medium, and hard. In the easy condition, the Tetris pieces would fall at a slow pace, with no possibility for the player to accelerate the fall. In the medium condition, the difficulty level or falling speed of the Tetris pieces was adjusted during the game as a function of the player’s performance. Finally, in the hard condition, the speed was maintained so high that it was difficult to even clear one line, putting the player at risk of quitting. In Núñez Castellar et al. (2019), the level of the game was adapted as a function of the participant’s performance in the “medium” flow condition, while in the other two conditions, the participant just repeatedly played the game’s easiest or hardest level, respectively. In the same vein, Klarkowski et al. (2016) had participants play a session of *Left 4 Dead 2* against enemies that provided no resistance, against enemies that dynamically adapted their difficulty to the player’s skills, or against enemies that made the game close to impossible to play. Although not using a game, Ulrich et al. (2016) had participants complete an arithmetic task that involved summing the numbers presented on a screen in three difficulty conditions: a very easy one, a second one where the difficulty was dynamically adapted to the participant’s level, and a third one where the difficulty was clearly above the participant’s skill level.

While this first strategy ensures that challenge in the medium-difficulty flow condition is matched to the skills of the participant, it leaves a number of key differences between this condition and the easy or hard fixed condition. In particular, the task difficulty in the medium condition is adaptive and individualized, while it is not in the other two. It would seem preferable not to confound flow with the use of closed-loop adaptive algorithms, which have been shown to engage brain circuits in a rather unique way (Mishra & Gazzaley, 2014). Another uncommon implementation of this approach can be found in de Manzano et al. (2010) and Ullén et al. (2010), who invited professional pianists to play two pieces of classical music. Participants were asked to bring a piece of their own choice that they knew they could play well and enjoyed playing, while the other piece was selected by the researchers to be extremely difficult, even for professional pianists. This latter way of proceeding suffers, however, from other confounds such as differences across conditions in memory content, emotional value, or familiarity, to cite a few, as well as the possibility that participants may not choose a piece that is actually matched to their skills due to motivational considerations (such as fear of failure). This method thus appears equally problematic.

The second approach is to determine the level of the player before the experiment occurs and set the difficulty of the compared conditions accordingly. This approach typically attempts to contrast an optimal challenge, high-flow condition to low-flow conditions induced through either easy or hard challenges, but does not use adaptive algorithms during the experiment. This is what Harmat et al. (2015) did using Tetris. In a short game session before the flow induction experiment, the participant’s optimal level was determined by having the participant play Tetris for 6 minutes in adaptive mode. The level achieved at the end of the training period was taken as the optimal flow level for that participant. Then the levels for the easy and hard challenge conditions were determined as being three levels above the optimal level (frustration) and three below the optimal level (boredom), respectively. Using the same game, de Sampaio Barros et al. (2018) implemented a slightly different method, whereby the easy Tetris condition was set at the easiest difficulty level of the game, the medium flow level was determined beforehand as the level at which the participant could make four lines with 30 pieces, and the hard level was implemented by doubling the falling speed of the medium level. Finally, Tozman et al. (2017) invited national- and international-level chess players with an Elo ranking to play a game of chess at the lab. Participants had to play either against a computer at their Elo ranking level or against opponents 400 Elo above or below their level, which corresponds to a 10% or 90% chance of winning, respectively.

Overall, these three studies were successful at inducing a higher flow state in the optimal challenge condition than in the easy (often termed boredom) or hard (often termed frustration) conditions. Of note, two of these studies also investigated the physiological correlates of flow using cardiac and respiratory measures. While Harmat et al. (2015) did not find any difference between their conditions in terms of heart rate or its variability, Barros and collaborators (2018) observed increased heart rate with increasing difficulty, but no difference in heart rate variability. Given their low cost and ease of use, whether physiological measures, such as heart rate and heart rate variability, can be relied upon as online measures of the flow state remains an interesting avenue to explore. In Experiments 2 and 3, we investigated how peripheral physiological measures may be used to provide objective markers of flow.

The present work builds upon these studies and the goal of identifying online markers of the flow state. To this end, we propose to manipulate flow by comparing two conditions with different levels of challenge carefully chosen to induce low and high levels of flow while ensuring that participants remain on task, i.e., try their best in both conditions. In this way, the present study furthers recent flow studies that have rather contrasted their optimal challenge condition with other conditions that differ in their incentive to stay on task because their level of difficulty is set to induce either boredom or frustration. In doing so, participants may be more likely to disengage from the task in the easy or hard conditions than in the high-flow condition of interest. In the present study, we propose controlling that participants stay similarly on task in a low-flow baseline condition and high-flow condition of interest.

Hence, the present work introduces a method to reliably generate two key experimental conditions to reveal possible neural or physiological markers of flow, while controlling for matched on-task behavior. As reviewed above, a first methodological choice is to contrast conditions that induce more or less flow while limiting possible confounds such as differences in adaptive procedure or in familiarity across conditions. Our approach will thus closely mirror that of Harmat et al. (2015) in terms of conditions contrasted, and in particular avoid contrasting one condition with a closed-loop adaptive mechanism to one without. Unlike Harmat et al. (2015), our experimental design includes two conditions instead of three: one where the challenge is matched to the skills of the participant and thus the probability of experiencing flow state is high, and a second one of greater challenge where the probability of experiencing flow state is lower. Importantly, these two conditions were designed such that participants remained on task and kept trying their best in each of them. To control that this was indeed the case, we tracked how much participants tried their best in each condition using the Effort/Importance subscale of the Intrinsic Motivation Inventory, which includes items such as “*I did my best while I was playing the game”* (see SM-02 in the supplementary materials for the full list of items)*.* After establishing this method, we ask whether such different flow states induce different levels of peripheral physiological activity using cardiac, respiratory, and electrodermal recordings.

## Experiment 1

Experiment 1 aimed at developing a procedure to induce low and high levels of flow level between two comparable on-task conditions. We devised a procedure to induce either high or low levels of flow by manipulating the challenge/skill balance within a video game, while at the same time ensuring that participants stayed on task, trying their best in each condition. To this end, we used the video game *Unreal Tournament* 2004 (*UT2004*), a first-person shooter game that provides the typical high-pace, absorbing, and challenging type of gameplay beneficial for maintaining flow. The game also provides a straightforward measure of performance computed as the kill-to-death ratio (KDR) within a level, and offers the possibility to finely manipulate difficulty by tuning the skill and the number of artificial intelligence (AI) opponents. We present an individualized procedure to tailor the difficulty of the game to the participant’s skills during a training phase using the KDR as a performance criterion. This approach was used to generate two experimental conditions: one “high-flow” condition where difficulty was matched to the participant’s skills, and one “low-flow” condition where difficulty was too high for the participant but still within a reasonable range to avoid having them give up the game.

Our procedure first involved finding the level of the game to induce flow given the participant’s skills, using the same game mode and map for each participant. We operationalized this level as the level where participants would have a KDR between 2 and 0.5, meaning players were at most twice as likely to kill than be killed (KDR = 2) or to be killed than killing (KDR = 0.5). Within this KDR range, players roughly achieve as many kills as deaths, a sign that the skills of their opponents (i.e., the challenge of the task) are balanced to their own skills. Once this level was determined for a participant, it was set as the high-flow level for this participant. The low-flow condition was then created by increasing the game difficulty by three levels (on a scale from 1 to 8 preset difficulty levels in the game). By matching skills to game challenges for each participant, we optimized the probability that flow would emerge in the high-flow condition. In contrast, by increasing the challenge above the participant’s skill level by a systematic value, we made sure that all participants experienced the same contrast in challenge between their high-flow and low-flow conditions. Crucially, this increase in difficulty was set to keep the game within a playable range for a wide variety of participants, ensuring that participants kept trying their best at the task even if somewhat frustrated.

To measure flow, we used the Flow State Scale (Jackson & Marsh, 1996) that assesses, through separate subscales, the nine components of flow described by Nakamura and Csikszentmihalyi (2014): *challenge-skill-balance, unambiguous feedback, clear goals, concentration, sense of control, action-awareness merging, transformation of time, loss of self-consciousness,* and *autotelic experience* (which refers to the phenomenon that the activity is experienced as enjoyable and intrinsically rewarding). Given our aim to manipulate flow while having participants trying their best in both conditions, we used the Effort/Importance subscale from the Intrinsic Motivation Inventory (IMI; Deci et al., 1994) to check that participants similarly tried their best in both conditions. This subscale asks “how hard participants tried to do the task,” with the exact phrasing of the four questions we used for this scale being “*I did my best while I was playing the game*,” “*I put a lot of effort into playing the game*,” “*I tried very hard on this game*,” and “*It was important to me to do well at this game*.” We refer to this subscale as *“*Best-Try*”* rather than “effort” to prevent the possible confusion that may arise from the different definitions of effort by other motivation frameworks (see Experiment 2 [E2]). Second, we used the *Perceived Competence* subscale from the IMI to check that our experimental manipulation of challenge successfully impacted participants’ perceived competence at the task. We expected perceived competence to be higher in the high-flow condition, where skills and challenges are matched, than in the low-flow condition, where difficulty exceeds skills. Third, we also used the *Interest/Enjoyment* subscale of the IMI to check that interest remained high across our two conditions, even if possibly lower in the low-flow than the high-flow condition. Finally, we used as a more exploratory measure the *Pressure/Tension* subscale of the IMI to assess how tense and pressured participants felt while playing. Given that our experiment did not involve performance-contingent incentives (e.g., a given goal to reach in the game or monetary rewards linked to performance), external pressure, or a social component, we expected this scale to remain rather low across both conditions, with possibly greater pressure/tension in the low-flow, more difficult condition.

The primary goal of Experiment 1 is to present a procedure to induce in each participant a high-flow state versus a low-flow state while controlling for best-try, as defined above. This novel contribution is outlined in detail below in the section “Individualized procedure to titrate high-flow and low-flow experiences.” Here our hypotheses were as follows and will remain the same for all three experiments: (i) the Flow State Scale (FSS) score will be higher in the high-flow (matched challenge) condition than in the low-flow (over-challenging) condition; (ii) the best-try score will be similarly high in the high-flow (matched challenge) and the low-flow (over-challenging) condition.

A secondary, pragmatic goal of Experiment 1 was to assess the possibility of inducing such flow variations while playing the game without the associated soundtrack. Indeed, it has been previously suggested that in-game sounds may have a positive influence on the emergence of flow (Nacke et al., 2010). However, with an eye toward using this paradigm to track the neural bases of flow, and given our aim to port it to a magnetic resonance imaging (MRI) scanner (Experiment 3), it was important to check the role of sound stimulation, if any, in our flow manipulation. As such, we also checked that our flow manipulation would be effective even without in-game sound.

### Materials and methods

#### Participants

Twelve healthy individuals—11 men and 1 woman between 18 and 32 years of age (mean age = 22, *SD* = 2.9 years)—participated in this study. They were covertly recruited from the Bavelier lab pool of participants. Each participant in this pool has filled out a series of questionnaires, including one about their video game play habits, and has been asked to play in the lab at least one 10-minute session of the game *Unreal Tournament* 2004 (*UT2004*). For each play session, the number of kills and deaths during the match were recorded.

Participants were recruited based on the following inclusion criteria:

1. Their familiarity with first- or third-person shooter (FPS) games: Participants had to have played on average 1 to 10 hours of shooter games per week over the past year. This ensured that participants were already familiar with the basic game play mechanics of shooters.
2. Their score during their in-lab 10-minute play: Participants’ kill-to-death ratio (KDR, number of kills over number of deaths) in Unreal Tournament during our screening procedure had to be between 0.5 and 4. In this sample, all participants completed two sessions. We utilized the KDR obtained from their second session, as it provides a more accurate estimate of their skill level in the game, given that participants typically acclimate to the game during their initial session.
3. Their age: Participants had to be between 18 and 35 years old.
4. Finally, all participants had to be right-handed and to have normal or corrected-to-normal vision.

The sample size for this and the following two experiments could not be based on power computations, as the expected effect size was unknown given the aim of establishing the feasibility and robustness of the novel methodology we present.

Participants were given information about the experiment and were asked to provide informed consent in accordance with the Declaration of Helsinki, under a protocol approved by the ethics commission of the Faculty of Psychology and Educational Sciences of the University of Geneva, and were paid for their participation.

#### Experimental design

All participants were asked to play *UT2004* successively in four different conditions, crossing the factors of flow level (high, low) and sound (with sound, no sound). In the high-flow conditions, participants played a free-for-all deathmatch against four other players controlled by the computer (called bots) at a level that was adapted to their skill (i.e., the challenge matched their skill), while in the low-flow condition, they played a free-for-all deathmatch against four bots that were too skilled for them (i.e., the challenge exceeded their skill). Participants played at each level twice, once with game sounds activated and once with them deactivated. The 12 participants were assigned to one of four orders counterbalancing the flow and the sound factors, such that the first two games had sound on and the last two games had no sound or vice versa. Whether low or high flow was then presented first was counterbalanced, keeping the same order for sound and no-sound runs. Condition orders were interleaved across participants, with participant 1 performing the experiment in order 1, participant 2 in order 2, participant 3 in order 3, participant 4 in order 4, and back to 1 for participant 5, and so on until all participants had been recruited.

Since each player’s skill is unique, we developed a novel procedure to determine the level of the game that matches the skills of each participant. This individualized procedure is described in detail before the results section.

#### Materials and apparatus

##### Flow State Scale (FSS; Jackson & Marsh, 1996)

Flow was measured using a French translation of the Flow State Scale (FSS, Jackson & Marsh, 1996). As no agreed-upon translation existed at the start of our study, we share in the supplementary materials (SM-01) the French translation we developed. Note that participants were presented with the French translation of each item and its original English version in italicized font just underneath. This 36-item questionnaire assesses each of the nine components of flow described by Csikszenmihalyi (Nakamura & Csikszentmihalyi, 2014) using one subscale for each component. Each subscale comprises four items evaluated on a five-point Likert scale. For example, for the component of flow *sense of control*, participants evaluate the statement “*I had a feeling of total control*,” or for the component *concentration* they evaluate the statement “*I was completely focused on the task at hand*.” The nine components of this scale are known to have good internal consistency, with Cronbach alphas between 0.80 and 0.86 on a sample of 394 participants (Jackson & Marsh, 1996). We report the Cronbach alphas for our studies in the supplementary materials (SM-01). Flow is measured as the average points over all 36 items.

##### Intrinsic Motivation Inventory (IMI; Deci et al., 1994)

We used a four-item subset of each subscale to keep the questionnaire as short as possible. Our selection of four items was based on the authors’ statement that “the incremental R for every item above 4 for any given [subscale] is quite small,” and the results of a validity study by McAuley et al. (1989) who also used four items per subscale. As in the original IMI, all the items in the IMI were rated on a seven-point Likert scale from “*not at all*” to “*very true*.” Our reduced IMI version can be found in full in the supplementary materials along with the Cronbach alphas for each subscale (SM-02). As no agreed-upon translation existed at the start of our study, we share in the supplementary materials (SM-02) the French translation used. Similarly, as for all the scales in these studies, participants were presented with the French translation of each item and its original English version in italicized font just underneath.

##### Best-try (Effort/Importance subscale)

We based our selection of four items on a previous study that had used the IMI in a video game context (Vos et al., 2011) and the reliability analysis from McAuley et al. (1989). The selected items are “*I did my best while I was playing the game*,” “*I put a lot of effort into playing the game*,” “*I tried very hard on this game*,” and “*It was important to me to do well at this game*.”

##### Perceived competence

Perceived competence was measured using a four-item subset of the *Perceived Competence* subscale. We selected the four items based on a previous study that used the IMI in a video game context (Vos et al., 2011) and the reliability analysis from McAuley et al. (1989). The selected items are “*I think I am pretty good at this game*,” “*I think I did pretty well at this game, compared to other participants*,” “*I was pretty skilled at this game*,” and “*I think I was good at this game*.”

##### Interest

Interest was measured using a five-item subset of the *Interest/Enjoyment* subscale of the IMI. We selected the four items from a previous study that used the IMI in a video game context that showed the best reliability in a previous reliability analysis (McAuley et al., 1989). The items in this subscale were “*I enjoyed playing this game very much*,” *“This game was fun to play*,” “*I thought this game was quite enjoyable*,” and “*I would describe this game as very interesting*.” We also added the item “*I thought this was a boring game*” given the importance of avoiding boredom.

##### Tension

Tension felt by the participant during the game was measured using a four-item subset of the *Pressure/Tension* subscale of the IMI. We selected the four items from the McAuley et al. (1989) study that had the best reliability. The items included are “*I felt pressured while playing the game*,” *“I felt very tense while playing the game*,” “*I was very relaxed in playing the game*,” and “*I was anxious while playing the game*.”

##### Objective difficulty measure

The kill-to-death ratio (KDR) was initially used in our procedure to assess a participant’s skills during the training phase and to titrate the level of challenge of the high-flow and low-flow conditions during the subsequent main experiment. KDR was also monitored during the main experiment as an objective measure of difficulty in both conditions and used as a sanity check to ensure that the two conditions presented different levels of challenge to the participant. We log-transformed the raw KDR given that the difference between a KDR of 0.1 and that of 0.5 is the same as that between a KDR of 2 and a KDR of 10 (both reflect that one game had a difficulty level five times greater than the other).

#### Individualized procedure to titrate high-flow and low-flow experiences

Our procedure aimed for a balance between skills and challenge, avoiding a situation in which participants either never die (too easy) or die so fast that it is frustrating. Accordingly, we aimed for a KDR between 0.5 and 2 to titrate our high-flow setting. The low-flow level was then set at three levels higher than the high-flow level—for example, if the high-flow level was 4 (in-game name: *skilled*), then the corresponding low-flow level was 7 (in-game name: *inhuman*). The maximum level in the game is 8 (in-game name: *godlike*).

**Fig. 1:**
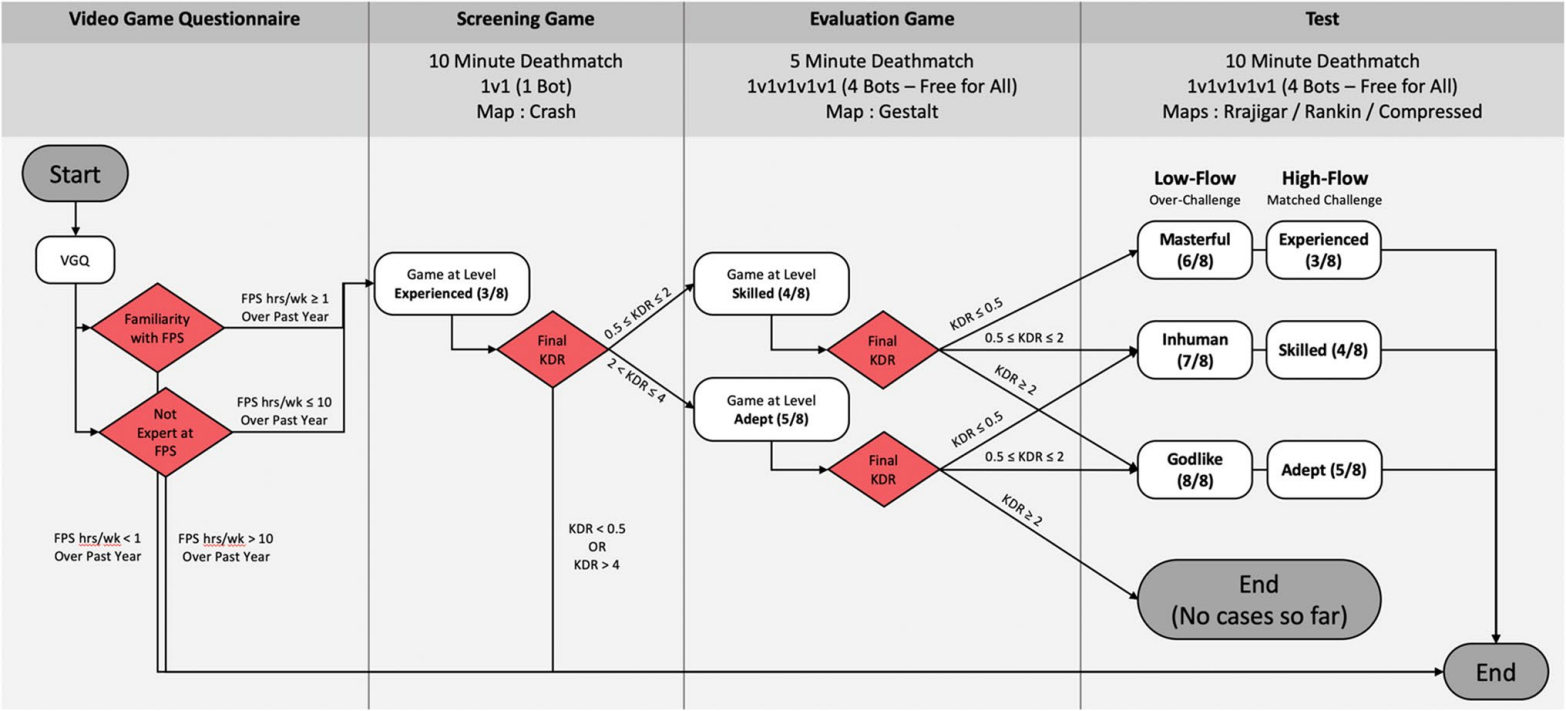
Flowchart of the procedure used to determine the difficulty of the game at which a participant is optimally challenged. For example, if a participant had a KDR of 0.86 during the screening game, then the bot skill in the evaluation game would have been set to level 4 (“*skilled*”). Then, if the participant had a KDR of 0.42 at the end of the evaluation game, the high-flow bot skill would have been set to level 3 (“*experienced*”) and the low-flow bot skill to level 6 (“*masterful*”). No participant had a KDR higher than 2 during the evaluation game

To find the high-flow setting, this procedure built on two different *UT2004* game play episodes: a 10-minute screening game play and 5-minute evaluation game play. The screening game consisted of a 1v1 deathmatch against a bot (a player controlled by the computer) with the difficulty level set on *experienced* (3 out of 8) and played on the map *Crash*. The evaluation game was a free-for-all deathmatch with four bots, using the *Gestalt* map, at a difficulty level selected according to their KDR at the outcome of the prescreening. As presented in Fig. 1, if their prescreening KDR was equal to or less than 2, the evaluation game was set on the *skilled* mode of *UT2004*. If their prescreening KDR was more than 2, the evaluation game was set on the *adept* mode of *UT2004*. All other parameters were similar to those of the other games as listed below. The experimenter then recorded the KDR of the participant and determined their high-flow and low-flow levels according to the procedure shown in Fig. 1.

If the participant played the evaluation game in *skilled* mode and their KDR was equal to or lower than 0.5, then their high-flow level was set to *experienced* (3 out of 8, or 3/8) and their low-flow level to *masterful* (6/8). If their KDR was between 0.5 and 2, then their high-flow level was set to *skilled* (4/8) and their low-flow level to *inhuman* (7/8). If the participant played the evaluation game in *adept* mode and their KDR was equal to or lower than 0.5, then their high-flow level was set to *skilled* (4/8) and their low-flow level to *inhuman* (7/8). If their KDR was between 0.5 and 2, then their high-flow level was set to *adept* (5/8) and their low-flow level to *godlike* (8/8). No player reached a KDR greater than 2 during their evaluation game.

#### Procedure

**Fig. 2:**
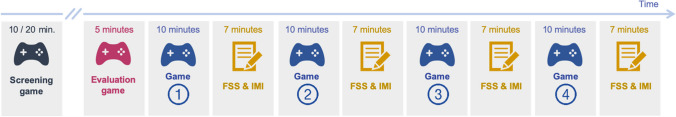
Experimental protocol for Experiment 1. Before the experiment presented here, participants underwent a screening where, among other tasks, they played at least one 10-minute screening game on *UT2004* (“screening game”). In the current study, participants also first played a 5-minute “evaluation game” using *UT2004* to determine their optimal level at the time of the experiment. The main experiment consisted in participants playing four 10-minute rounds of *UT2004* in each of the four conditions described above (high/low-flow × sound/no-sound), with the order semi-counterbalanced across subjects

Participants were given information about the experiment and were asked to provide informed consent. They were then asked to go to an isolated soundproof booth and were given a brief overview of the specifics of *UT2004* (for instance, what each weapon does or what health packs look like). The participants then played a 5-minute evaluation game (as described in our titration procedure), and the difficulty levels for their high-flow and low-flow games were determined. The participants then played four 10-minute games, crossing the factors flow (high flow vs. low flow) and sound (present vs. absent)*.* The map was different for all four game sessions to avoid confounds from knowing the map better from one game to the other, but followed the same order for all participants (*Rankin* / *Rrajigar* / *Corrugation* / *Compressed*). A summary of the procedure is given in Fig. 2. The parameters for the game can be found in the supplementary materials (SM-09).

At the end of each 10-minute game session, the participants first completed the FSS and then the subset of questions from the IMI, with the instruction to answer these questionnaires based on their experience during the *last game only*. After completing these two questionnaires, they were allowed to take a short break before proceeding with the next game. The participants saw the experimenter set the difficulty of game play and were instructed to just enjoy the game and perform as well as possible, even when the difficulty was too high for them. Each time the participants were playing, they were left to play alone in a dedicated room. In both the sound and no-sound conditions, participants were instructed to wear headphones. The questionnaires were filled out on the same computer as the game play was performed using a *LimeSurvey* interface.

### Results

The goal of Experiment 1 was twofold: (i) to evaluate the ability of our proposed procedure to manipulate flow state, expecting higher FSS in the high-flow than the low-flow condition, while keeping best-try matched across conditions, and (ii) to test whether the presence or absence of sound would have an impact on the flow manipulation.

Because we were also interested in quantifying the amount of evidence for the null hypothesis (or in other words, an absence of difference in best-try between the high-flow and low-flow conditions), we opted to use Bayesian statistical analyses. A small guide on how to interpret results from Bayesian ANOVAs and *t*-tests can be found in the supplementary materials (SM-07). In addition, we report the results of the frequentist repeated-measure ANOVA on the FSS for all experiments in the supplementary materials (SM-08) as means of comparison with the results of the Bayesian analyses.

Using JASP software (JASP Team, 2018), we performed a Bayesian repeated-measure ANOVA with flow (*high flow* or *low flow*) and sound (*sound* or *no sound*) as within-subject factors on all our dependent variables, namely the flow measure from the *FSS* and the *best-try* measure*,* as well as the measures of *perceived competence, interest,* and *tension*. Since there might be differences between participants who started with a *high-flow* game compared to a *low-flow* game, we also added an “*order*” between-subject factor.

**Fig. 3:**
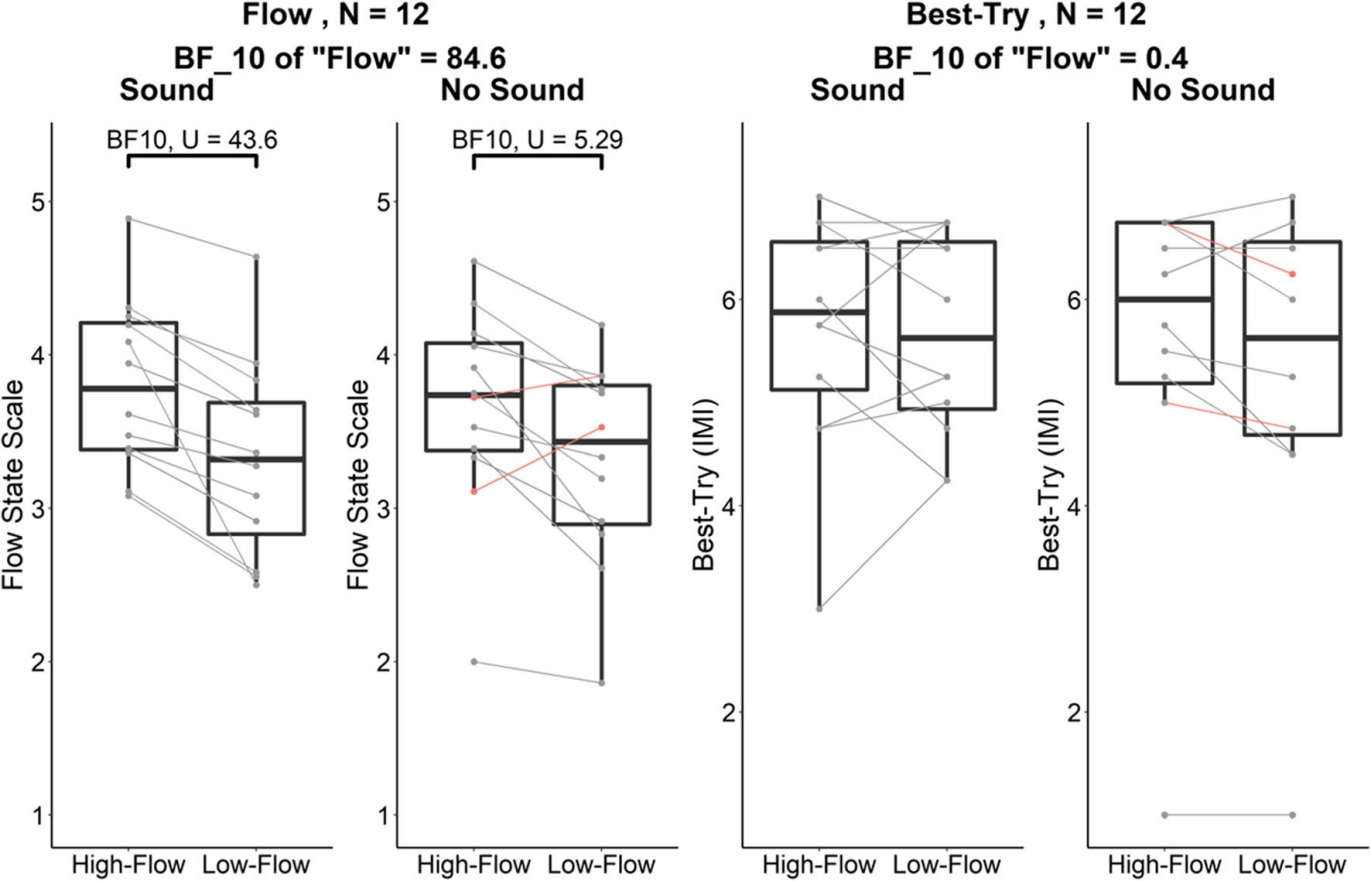
Experiment 1. Boxplots of the individual scores on the Flow State Scale (FSS- two plots on the left) and Best-Try scale (two plots on the right) in the high-flow and low-flow conditions with and without sound on. Each boxplot (and all the following) represents the median and interquartile interval; stars are outlier values (more than 1.5 times the interquartile interval away from the median). The individual scores are superimposed and linked by a line whose color of the links displays whether the difference in FSS between high-flow and low-flow conditions was positive (gray—as expected) or negative (red)

The data for the FSS and the Best-Try scale are presented in Fig. 3. Descriptive statistics for all the dependent measures in all four conditions are given in Table 1.

**Table 1:** Experiment 1. Descriptive statistics (*N* = 12). For each dependent measure, average and standard deviation in parentheses are reported. The evidence for an effect of flow condition is the Bayes factor of the model containing only the main effect of flow against the null model. FSS, Flow State Scale; Perceived comp., perceived competence. Non-log-transformed KDR values are given for information, as they are more straightforward to interpret than the log-transformed values used in the analyses

	High flow, Sound	Low flow, Sound	High flow, No sound	Low-flow, No sound	Evidence for an effect of flow condition
*Predicted to differ*					
FSS	3.81 (0.56)	3.33 (0.65)	3.66 (0.68)	3.31 (0.66)	BF_10_ = 84.6
*Predicted to be matched*					
Best-try (IMI)	5.73 (1.15)	5.68 (0.98)	5.6 (1.61)	5.4 (1.68)	BF_10_ = 0.4 = 1/2.5
*Variables measuring our challenge manipulation*					
Perceived comp. (IMI)	4.50 (1.68)	2.28 (0.86)	4.67 (1.62)	2.44 (1.17)	BF_10_ = 3.9×10^6^
Log_10_(KDR)	0.071 (0.20)	−0.61 (0.22)	0.083 (0.20)	−0.60 (0.18)	BF_10_ = 1.9×10^14^
KDR	1.18	0.25	1.21	0.25	
*Additional motivational variables*					
Interest (IMI)	5.22 (1.68)	4.46 (1.46)	4.96 (1.41)	4.56 (1.32)	BF_10_ = 2.5
Tension (IMI)	3.43 (1.18)	3.86 (1.48)	3.23 (1.35)	3.5 (1.64)	BF_10_ = 0.6 = 1/1.67

#### Flow score (FSS)

The Bayesian repeated-measure ANOVA with *flow* and *sound* as within-subject factors and *order* as a between-subject factor (Table 2) indicated very strong evidence for the model with only the main effect of *flow* on FSS score (BF_10_ = 84.6) compared to the null model, and anecdotal evidence against the model with only the main effect of *sound* (BF_10_ = 0.34 = 1/2.94) or *order* (BF_10_ = 0.61 = 1/1.64) compared to the null model. In addition, adding the interactions did not improve the models.

**Table 2:** Experiment 1. Model comparisons for the FSS score. Results of the Bayesian repeated-measure ANOVA (JASP) with flow and sound as within-subject factors and order as a between-subject factor (we show here only the 9 models including flow out of the 19). The best-fitting model is the one with flow as a unique factor (in bold). P(M|data) denotes the posterior probability of the listed model, BF_10_ is the Bayes factor (or evidence) of the model against the null model. We used a uniform prior distribution

Models	P(M)	P(M|data)	BF_10_
Null model (incl. subject)	1/19	0.003	1.000
**Flow**	**1/19**	**0.219**	**84.586**
Sound	1/19	8.770e−4	0.338
**Flow** + sound	1/19	0.080	31.034
**Flow** + sound + **Flow** ✻ sound	1/19	0.036	13.836
Order	1/19	0.002	0.610
**Flow** + order	1/19	0.146	56.491
**Flow** + order + **Flow** ✻ order	1/19	0.039	14.915
**Flow** + sound + **Flow** ✻ sound + order + Flow ✻ order + sound ✻ order + Flow ✻ sound ✻ order	1/19	0.009	3.657

*Note.* All models include subject

#### Best-try (IMI – Effort/Importance)

In line with best-try being matched across flow conditions, there was anecdotal evidence against the model with only the main effect of *flow* on best-try (BF_10_ = 0.38 = 1/2.63), anecdotal evidence against the model with only the main effect of *sound* (BF_10_ = 0.50 = 1/2), and anecdotal evidence for the model with only the main effect of *order* (BF_10_ = 1.41) compared to the null model. In addition, adding the interactions did not improve the models.

#### Perceived competence (IMI – Perceived Competence)

As expected, there was decisive evidence for the model with only the main effect of *flow* on perceived competence (BF_10_ = 3.89×10^6^) compared to the null model, and weak evidence against the models with only the main effect of *sound* (BF_10_ = 0.29 = 1/3.45) or *order* (BF_10_ = 0.32 = 1/3.13) compared to the null model. In addition, adding the interactions did not improve the models.

#### Objective difficulty (Log_10_(KDR))

As expected, there was decisive evidence for the model with only the main effect of *flow* on Log_10_(KDR) (BF_10_ = 1.95×10^14^) compared to the null model. As for perceived competence, there was weak evidence against the models with only the main effect of *sound* (BF_10_ = 0.29 = 1/3.45) or *order* (BF_10_ = 0.19 = 1/5.26) compared to the null model. In addition, adding the interactions did not improve the models.

#### Interest (IMI – Interest/Enjoyment)

There was anecdotal evidence for the model with only the main effect of *flow* on interest (BF_10_ = 2.5), and weak evidence against the models with only the main effect of *sound* (BF_10_ = 0.29 = 1/3.45) or *order* (BF_10_ = 0.43 = 1/2.33) compared to the null model. In addition, adding the interactions did not improve the models.

#### Tension (IMI – Pressure/Tension)

There was anecdotal evidence against the models with only the main effect of *flow* on tension (BF_10_ = 0.65 = 1/1.54), *sound* (BF_10_ = 0.46 = 1/2.17), or *order* (BF_10_ = 0.62 = 1/1.61) compared to the null model. In addition, adding the interactions did not improve the models.

### Discussion: Experiment 1

Experiment 1 confirmed that our adaptation of difficulty to individual skills in *UT2004* was effective at changing the state of flow as measured with the FSS. The effect size of the difference in flow between the high- and low-flow conditions was 1.36 (Cohen’s *d*; 95% CI [0.55, 2.14]), reflecting a strong effect with a mean difference of 0.41 (*SD* = 0.33).

Experiment 1 also confirmed that this manipulation in flow level can be achieved in the absence of the game sounds. Participants played *UT2004* at the same level of difficulty with or without the game sounds, always keeping the headset on. Our flow manipulation (difference in FSS between the high- and low-flow conditions) was numerically smaller without sound (0.35) than with sound (0.48). This finding is also reflected in two subjects reporting greater FSS scores in the low-flow than in the high-flow condition without sound, but none when the sound was on. However, our results yielded inconclusive evidence for or against a main effect of sound or an interaction of sound with flow. Evidence for a difference between high-flow and low-flow conditions when the participants played without sound was observed with an effect size of *d* = .87 (95% CI [0.19, 1.53]). These results pave the way to using the proposed paradigm in settings where sound may not be available, as shown in the next two experiments.

We also note that our results are unlike those of Nacke et al. (2010), who reported lower flow in a no-sound condition than in a sound-on condition, using the flow dimension of the Game Experience Questionnaire (GEQ; IJsselsteijn et al., 2007; Poels et al., 2007). One attribute to monitor in future studies to better understand the impact of sound on flow may be the degree of video game expertise in the population sampled. Indeed, although measuring immersion rather than flow, Zhang & Fu (2015) reported that presence or absence of in-game sounds did not change immersion in habitual video game players (about 33 hours/week), in contrast to participants playing fewer hours per week (7 hours/week), for whom in-game sounds increased immersion. In line with that proposal, our sample comprised participants playing video games on average 11 hours/week (range [4;31]), with all participants playing more than 2 hours/week of FPS, whereas Nacke et al. (2010) sampled from the general student population without criteria on video game play hours, possibly recruiting participants with less video game expertise. This effect might also be mediated by game type. For example, Wiemeyer, (2013) reported no difference in flow (measured by the flow construct of the GEQ) in exergames in a sample of habitual and non-habitual video game players. Exergames differ, however, in terms of theme, situations to overcome, challenges, and attentional load compared to the first-person shooter games used in Nacke et al. (2010); Zhang & Fu (2015). Whether video game usage habits and game type may explain different sensitivity of flow to in-game sound remains to be fully addressed.

Finally, while a clear difference in flow was observed between high- and low-flow conditions, our Bayesian analyses indicated that the degree to which participants tried their best was relatively comparable across those conditions, with anecdotal evidence for the null (BF_10_ = 1/2.63). Our novel paradigm achieved this by individually tailoring the game play level to reach a balanced KDR for each participant and systematically going three levels higher than this personalized high-flow level to generate a more difficult, but not overwhelmingly so, low-flow condition. Only two other flow studies have used a similar procedure when using video games to induce flow (de Sampaio Barros et al., 2018; Harmat et al., 2015); importantly, the present study is the first to assess whether best-try behavior is maintained when inducing high- and low-flow conditions with such a procedure.

As predicted, presenting a higher play challenge than the participant’s skill level resulted in lower objective ease of play (KDR) and perceived competence of participants in the low-flow versus high-flow condition.

Finally, we expected similarly high and matched measures of interest as well as similarly low and matched measures of tension. We did observe similarly high above-average interest and similarly low below-average tension scores across the two flow conditions. This pattern of results suggests that our manipulation led to two conditions of relatively high interest without applying unnecessary tension on the participants in either condition.

Experiment 1 presented a procedure to systematically induce both low- and high-flow conditions, all the while ensuring participants stayed on task, as measured by participants equally trying their best (IMI Effort/Importance scale). In Experiment 2, we recruited a larger sample to achieve more conclusive levels of evidence in our main comparisons. We also wanted to investigate whether our so-induced states of low and high flow could be related to different states of sympathetic and parasympathetic activation.

## Experiment 2

Experiment 2 is a replication and extension of Experiment 1. First, to extend our understanding of the interplay between flow, motivation, and effort, we wished to address more directly how difficulty level affects the motivational state of the player, varying from flow to frustration or to boredom. Indeed, while participants showed greater performance (both subjective as reported by the IMI–Perceived Competence subscale, and objective as shown by the KDR), it is possible that the participants did not subjectively feel that one condition required more effort than the other. Thus, to disentangle the effect of our game difficulty manipulation on effort from that on best-try, we added to our battery of questionnaires the Rating Scale of Mental Effort (RSME; Zijlstra, 1993), where participants reported “*how much effort it took them to do the task they just completed*.” To date, only one study has directly investigated the link between flow and how much effort it took participants to play a game at easy, medium, and hard challenge levels (Harris et al., 2017). Using the RSME, the effort produced to complete the medium-challenge, high-flow condition (not tailored to the skills of the participant) was found to be higher than that for the easy condition and lower than that for the hard condition. Thus, RSME-based effort mirrored the difficulty level of the game, and not flow level. Similarly to Harris et al. (2017), we expected effort to change with task difficulty and to be higher in the low-flow than the high-flow condition.

We were also interested in how our measure of flow interacted with a measure of frustration. Indeed, while frustration may lead to the participant giving up on the task, it may also be an integral part of the flow experience, as flow can be experienced in those moments where one overcomes a difficult challenge, which necessarily elicits frustration (Csikszentmihalyi, 1990). In addition, in the video game design literature, it has been suggested that occasionally putting the player in purposefully designed frustrating situations leads to greater immersion in the game (Lyons, 2015; Qin et al., 2010), and observations from purposefully hard games tend to show that while the games can be extremely frustrating, high satisfaction can be derived from overcoming the challenges within these games (Birk et al., 2015). Two frustration questions were thus added as an exploratory measure on how to best control for motivational and effort constructs across our flow conditions.

Additionally, we collected cardiac (heart rate and high-frequency heart rate variability [HF-HRV]), respiratory (respiratory rate and depth), and electrodermal (skin conductance level [SCL]) measures in an effort to assess the physiological correlates of high- versus low-flow state. The most widely accepted model of the physiology of flow to date posits that flow is characterized by “moderate levels of sympathetic activation and moderate parasympathetic activation—i.e., a sympathetic-parasympathetic co-activation” (Peifer & Tan, 2021; Ullén et al., 2010). In contrast, in a low-flow condition such as ours during which challenge is higher than in the high-flow condition, increased sympathetic activation and decreased parasympathetic activation is expected. In terms of physiological measures, lower sympathetic activation in high flow than in low flow is expected to translate into decreased SCL (Boucsein, 2012) and decreased respiratory rate as well as increased respiratory depth and HF-HRV because of parasympathetic activation (Lumma et al., 2015; Wientjes, 1992), and decreased heart rate due to the sympatho-vagal balance favoring parasympathetic activation (Porges, 1995).

The literature remains mixed, however, on observing such a pattern. This co-occurrence of sympathetic and parasympathetic activation in the high-flow condition was reported by Harmat et al. (2015), who found lower respiratory depth in their lower-flow, more demanding condition as compared to high-flow condition. We note, however, that a variety of effects have been reported in this burgeoning literature—for example, decreased sympathetic with similar parasympathetic activation in the high-flow as compared to the low-flow, difficult condition (de Sampaio Barros et al., 2018), increased parasympathetic activation in the high-flow condition compared to an easy and a hard condition (Tozman et al., 2015), increased sympathetic activation in the high-flow condition compared to easy and hard low-flow conditions (Ulrich et al., 2016, 2022), or even increased sympathetic activation and decreased parasympathetic activation in the high-flow condition compared to the low-flow difficult condition (Harris et al., 2017). In the present study, we expected measures reflecting parasympathetic activation (HF-HRV and respiratory depth) to be higher in the high-flow than in the low-flow more difficult condition, and both to be lower than during the rest condition. We predicted higher SCL, heart rate, and respiratory rate in the high- and low-flow conditions compared to rest. Given the mixed results observed in the literature, we did not have any strong hypothesis regarding SCL. In addition, because heart rate and respiratory rate are both influenced by the sympathetic and parasympathetic nervous system, differential prediction between flow conditions remains uncertain. These measures, however, will give us information on the sympatho-vagal balance when participants try their best equally between a high- and a low-flow condition.

Finally, Experiment 1 instructed participants to “just enjoy the game and perform as well as possible,” which may have pressured participants to report similarly high scores on the Best-Try scale in both conditions. To address this potential confound, the participants’ instructions before the experimental games (i.e., the games after the evaluation game has been completed) were modified to make no mention of enjoyment or performance, by stating, “We will ask you to simply play a match of *Unreal Tournament* against four other players controlled by the computer (also called bots). After each match, we will ask you to fill out a few questionnaires.”

### Materials and methods

#### Participants

Fifty-three healthy individuals participated in this study, but four of them had to be excluded due to technical difficulties. The final sample was thus composed of 49 participants—43 men and 6 women between 19 and 33 years of age (mean age = 23.25, *SD* = 2.98 years)—recruited using the same criteria as in E1.

Participants were given information about the experiment and were asked to provide informed consent in accordance with the Declaration of Helsinki, under a protocol approved by the ethics commission of the Faculty of Psychology and Educational Sciences of the University of Geneva, and were paid for their participation.

#### Design

The design was identical to that of E1 except for five main changes: (i) Participants were equipped with physiological sensors. (ii) Participants always played with sound off. (iii) We added a 5-minute resting state condition occurring a few minutes after the evaluation game but before the two 10-minute periods where the participants played *UT2004* at a high or low level of flow identically to E1. (iv) Among the questionnaires administered immediately after each game play period, the RSME and a question assessing frustration were added. (v) Another exploratory low-flow setting was added as an always-last 10 minutes of game play and will not be described further here. Finally, as noted in the introduction, a slight wording modification was made to the instructions to check whether our original wording could be at the source of the matched best-try.

#### Materials and apparatus

##### Questionnaires and skill measures

*Flow (FSS), motivation measures (best-try, perceived competence, interest, tension), KDR.* Same as in Experiment 1.

**RSME.** Changes in perceived effort to perform the task were assessed using the Rating Scale of Mental Effort (RSME; Zijlstra, 1993). It requires one to rate “*how much effort it took for [the participant] to play the game [they] just finished*” on a unidimensional visual scale ranging from 0 to 150, where 0 indicates “*absolutely no effort*” and 150 “*extreme effort*.” We share in supplementary materials (SM-03) the French version we used for this scale. As for the FSS and IMI subscales, the participants were presented with the French translation and its original English version side by side.

**Frustration.** Frustration was measured with two questions answered on a five-point Likert scale from 1 (a little) to 5 (a lot) adapted from the National Aeronautics and Space Administration (NASA) Task Load Index (TLX) questionnaire (Hart & Staveland, 1988), usually used to assess the subjective experience of a task. These items were presented in French; original versions are as follows: “*During the task, how insecure, discouraged, irritated, stressed and annoyed were you compared to being secure, satisfied, happy, relaxed and complacent?*” and “*How frustrated were you during the task?*” The French translation for these questions is reported in the supplementary materials (SM-04). Similarly to the other questionnaires, participants were presented each item with its French translation and its original English version in italicized font just underneath.

##### Physiological measures

Cardiac, electrodermal, and respiratory measures were all recorded using the Biopac MP150 system at 2000 Hz and AcqKnowledge 4.4 software.

**Heart rate and high-frequency heart rate variability.** An electrocardiogram (ECG) was recorded using a standard three-electrode setup with the positive electrode on the lower left ribs, negative electrode just below the right clavicle, and reference electrode just below the left clavicle. The electrodes were connected to a Biopac ECG100C module. Heart rate was measured as the inverse of the mean duration between two R peaks in the ECG, and high-frequency heart rate variability (HF-HRV) was measured as the power of the high-frequency band (0.15–0.4 Hz) normalized by the total power of the very low-frequency (0–0.04 Hz), low-frequency (0.04–0.15 Hz), and high-frequency (0.15–0.4 Hz) bands as computed in Kubios analysis software (Tarvainen et al., 2014, version 2.3). We used heart rate and HF-HRV as proxies for the sympatho-vagal balance and the parasympathetic activity, respectively (Task Force of the European Society of Cardiology and the North American Society of Pacing and Electrophysiology, 1996).

**Respiratory rate and depth.** Respiratory data were acquired using a Biopac breathing belt strapped around the lower part of the rib cage of the participant connected to a Biopac RSP100C module. The belt was fastened so that it did not impair breathing but was sufficiently tight that it would not slide down during the recording. Respiratory rate was measured as the inverse of the period between two peaks of successive respiratory cycles and respiratory depth as the mean amplitude of each respiratory cycle. Respiratory rate was used as a proxy for sympatho-vagal balance, and respiratory depth was used as a proxy for parasympathetic activation (Lumma et al., 2015; Wientjes, 1992).

**Skin conductance level.** The skin conductance level (SCL) was recorded using a pair of reusable Biopac GSR electrodes filled with conductance gel connected to a Biopac GSR100C module. The electrodes were located on the proximal phalanx of the index and the middle finger on the left hand of the participant. During play, this hand remained on the keyboard and was used to control movement in the game using the W, A, S, D keys. We made sure that the wires of the electrodes did not interfere with game play. Overall movement of the fingers during game play was limited enough that we were able to record electro-dermal activity with good reliability. We used mean SCL as a proxy for sympathetic activation (Boucsein, 2012).

##### Gaming equipment

Identical to E1.

#### Procedure

**Fig. 4:**
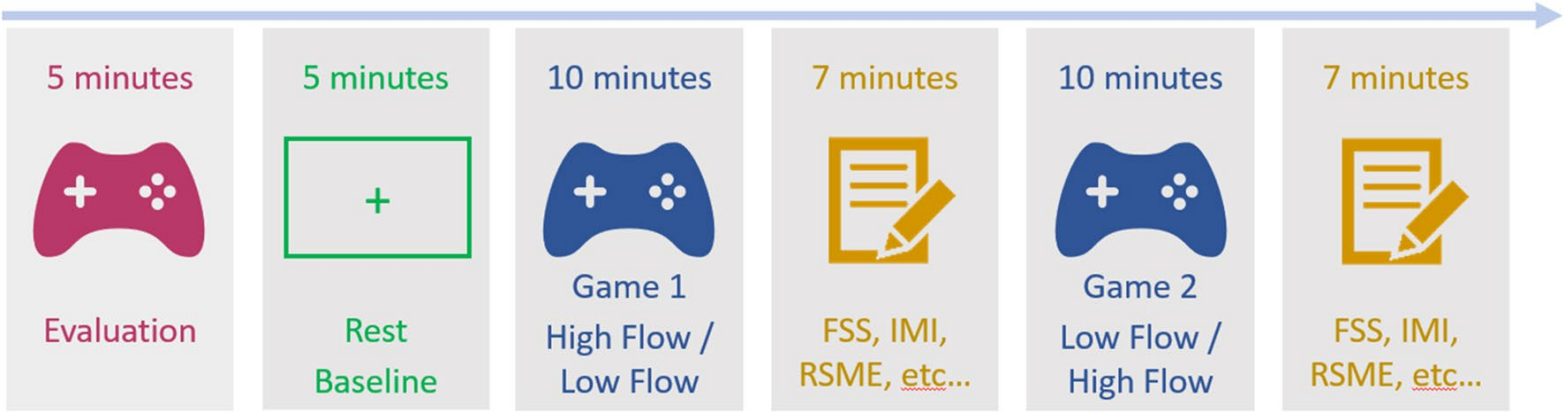
Experiment 2 study protocol showing time course of all analyzed conditions. The order of the low-flow and high-flow games was counterbalanced across subjects. The low-flow/easy-level condition was always done at the end of the experiment (not shown here). The screening game was done at a different time

Participants were first given information about the experiment and filled out the consent form. Then they were asked to go to an isolated soundproof booth and were equipped with the physiological recording equipment. They then completed the exact same 5-minute evaluation game as in E1. Game levels for the high- and low-flow conditions were then determined using the same procedure as in E1 (Fig. 1). This included using their KDR during a 10-minute screening game that occurred before the experiment (screening game in Fig. 3). Next, participants completed a 5-minute rest period, followed by the high-flow and low-flow game sessions, whose order was counterbalanced across subjects, and a final game in another low-flow setting which will not be further discussed. After each gaming session (high-flow and low-flow), participants were asked to complete the FSS, the IMI subscales (measuring *best-try, perceived competence, interest,* and *tension*), the RSME, and the questions on frustration as described in the “Questionnaires” section, in that order (Fig. 4).

- **Rest**: Participants were asked to fixate on a white cross (size: 1.3° visual angle, thickness: 0.18°) in the middle of a black screen for 5 minutes. They were told that they could think about whatever they wanted and were explicitly instructed not to close their eyes.
- **Games (high-flow vs. low-flow):** Same as in E1 except that participants were instructed as follows: “The experiment consists in three phases where we will ask you to simply play a match of *Unreal Tournament* against four other players controlled by the computer (also called bots). After each match, we will ask you to fill out a few questionnaires.” We also restricted the map selection to Rrajigar and Corrugation. The order of the low-flow and high-flow conditions and the order of the maps were fully counterbalanced across subjects. Subject attribution to each order was done in a similar fashion as in E1.
- **Questionnaires:** After the high-flow and low-flow conditions, participants completed the FSS, the four IMI subscales interleaving their items, the RSME, and the frustration questions in that order. These took from 6 to 10 minutes depending on the participant.

### Data analysis

For all physiological measures, raw data were first converted into MATLAB format using the default AcqKnowledge save function.

#### Cardiac measures (ECG)

Using the ECGdeli toolbox (Pilia et al., 2021), the data were first filtered using a low-pass filter at 120 Hz, a high-pass filter at 0.3 Hz, and a band-stop filter at 49–51 Hz and then isoline-corrected as implemented in ECGdeli. Data were then exported into Kubios (Tarvainen et al., 2014, version 2.3) for cleaning and computation of the cardiac measures. There we manually corrected for ectopic, missing, and extra R peaks, and then applied the “medium” level correction that “compares every IBI [inter-beat interval] value against a local average interval and removes IBIs that deviate by more than 0.25 s.” The corrected traces, heart rate, and HF-HRV were then saved for each participant. Finally, we log_2_-transformed HF-HRV values so that their distribution would follow a normal distribution. One extra subject was excluded from analyses for the ECG measures as electrodes did not stay in place properly during the experiment, giving a final sample size of 48 subjects for cardiac measures.

#### Respiratory measures

The signal was first down-sampled from 2000 Hz to 50 Hz to reduce computational load. Then, using EEGLab functions (Delorme & Makeig, 2004), the signal was band-pass-filtered between 0.05 Hz and 1 Hz to remove fast and slow artifact components, following the recommendation from Biopac^®^. Given the high inter- and intra-individual variability in respiratory amplitude and rate in our sample, we elected to use a semi-automatized method to identify the respiratory cycle period and amplitude. First, the MATLAB function *findpeaks* was used to identify the peaks in the filtered respiratory signal, with the constraint that two peaks be separated by at least 1.33 s (corresponding to 45 RPM). The time series of identified respiratory cycles were then visually inspected by two experimenters, and erroneous peaks or clear artifacts were removed from the cycle series and missed peaks were added. Finally, outliers in the period and amplitude time series were replaced by linearly interpolated values if they were more than three times the median absolute deviation (as implemented in the *filloutliers* MATLAB function). The respiratory rate was then computed as the mean of the inverse of the inter-peak intervals, and respiratory depth was measured by the average amplitude of the peaks across the session. Mean respiratory depth values were further log_2_-transformed so that they would follow a normal distribution.

Three subjects were excluded from respiratory rate analyses, one because their mean respiratory rate at rest was more than three times the scaled median absolute deviation from the median of our sample, and two because of issues with the breathing belt. For the respiratory depth analyses, we further excluded five participants whose breathing belt became loose during the experiment and saturated during inhalation, thus not properly recording the full amplitude of the respiratory cycle (but still providing reliable data for the respiratory rate, as the peaks were clearly identifiable). We thus had a final sample size of 46 for the respiratory rate analyses, and 41 for the respiratory depth.

#### Skin conductance level

The signal was first down-sampled from 2000 Hz to 50 Hz to reduce computational load. Then using EEGLab (Delorme & Makeig, 2004) functions, the signal was low-pass-filtered at 1 Hz to remove fast and slow artifact components, following recommendation from Biopac. Tonic and phasic components of the signal were extracted using the Continuous Decomposition Analysis as implemented in Ledalab (Benedek & Kaernbach, 2010). We then extracted the mean of the tonic component as the average SCL during each condition. We excluded seven extra participants from these analyses as they exhibited no response and did not show any phasic activity during the recording periods, thus giving us a final sample size of 42.

### Results: Behavior

A Bayesian repeated-measure ANOVA with flow condition (*high-flow* or *low-flow*) as within-subject factors was carried out on all dependent variables (namely *flow score [FSS], best-try, perceived competence, KDR, interest, tension, RSME, frustration*). As above, we also coded whether participants started with a high- or a low-flow game, with a between-subject “*order*” factor. The same guide as for E1 on how to interpret results from these ANOVAs can be found in the supplementary materials (SM-07). We also report the results of the frequentist repeated-measure ANOVA on the FSS in the supplementary materials (SM-08) as means of comparison with the results of the Bayesian analyses. Descriptive statistics for all the dependent measures in each condition are given in Table 3.

**Table 3:** Experiment 2. Descriptive statistics of the dependent behavioral measures (*N* = 49). The average is reported with the standard deviation in parentheses. The evidence for an effect of flow condition is the Bayes factor of the model containing only the main effect of flow against the null model. Non-log-transformed KDR values are given for information as they are more straightforward to interpret than the log-transformed values used in the analyses

	High flow	Low flow	Evidence for an effect of flow condition
*Predicted to differ*			
FSS	3.7 (0.35)	3.3 (0.43)	BF_10_ = 2.09e+07
*Predicted to be matched*			
Best-try (IMI)	5.7 (1.1)	5.7 (1.2)	BF_10_ = 0.23 = 1/4.35
*Variables measuring our challenge manipulation*			
Perceived competence (IMI)	4.2 (1.4)	2.5 (1.1)	BF_10_ = 1.42e+07
Log_10_(KDR)	0.19 (0.26)	−0.51 (0.3)	BF_10_ = 3.48e+20
KDR	1.25	0.30	
*Additional motivational and effort variables*			
Interest (IMI)	5.3 (1)	4.8 (1.1)	BF_10_ = 12.9
Tension (IMI)	3.2 (1.3)	3.8 (1.4)	BF_10_ = 94.8
Effort (RSME)	58 (20)	69 (25)	BF_10_ = 45.9
Frustration	2.3 (1.2)	3.2 (1.3)	BF_10_ = 892

#### Flow score (FSS)

**Fig. 5:**
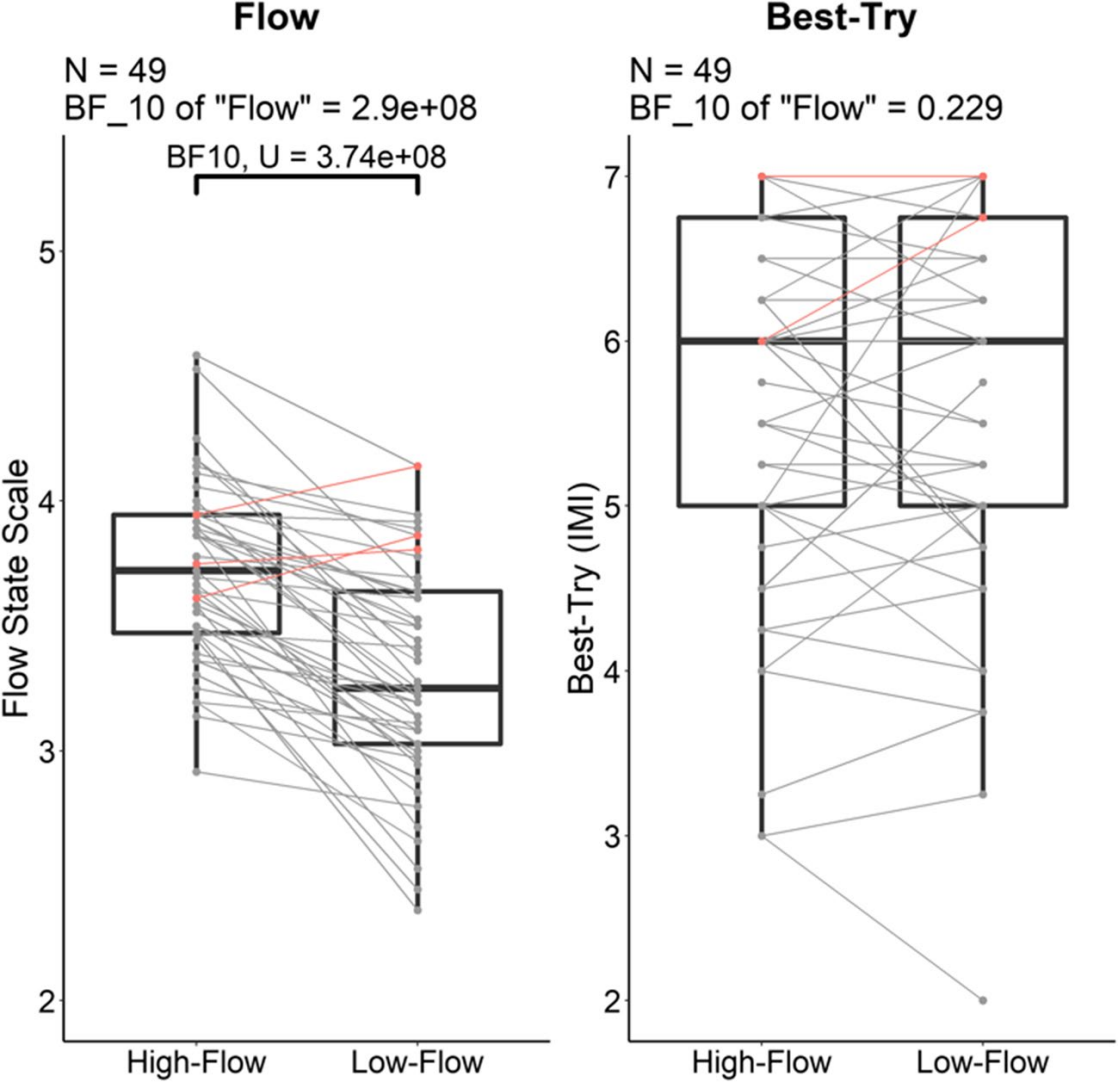
Experiment 2. Boxplots of the individual scores on the FSS (left) and the Best-Try subscale of the IMI (right) in the high-flow and low-flow conditions with the individual scores superimposed. The color of the links displays whether the difference in FSS score in the high-flow condition minus the FSS score in the low-flow condition was positive as expected (gray). Only three participants, shown in red, reported marginally higher FSS scores in the low-flow than in the high-flow condition. The evidence for the difference between our conditions is given as the BF_10,U_

The Bayesian repeated-measure ANOVA on the FSS score with *flow* (high vs. low flow) as within-subject factor and *order* as a between-subject factor indicated decisive evidence for the model including the main effects of *flow, order,* and the flow*order interaction (BF_10_ = 4.19×10^8^) compared to the null model. There was also smaller but decisive evidence for the model including only the main effect of *flow* compared to the null model (BF_10_ = 2.90×10^8^), and there was anecdotal evidence against the model with only the main effect of *order* (BF_10_ = 0.53 = 1/1.89; Fig. 5, left panel) compared to the null model. Further post hoc analyses showed that there was decisive evidence for adding the main effect of flow to the null model (BF_10,incl_ = 3.90×10^8^) and comparatively negligible evidence for adding either the main effect of *order* (BF_10,incl_ = 1.39) or the flow*order interaction (BF_10,incl_ = 3.52) to the null model.

#### Best-try (IMI – Effort/Importance)

In line with best-try being matched across flow conditions, there was weak evidence against the model with only the main effect of *flow* on best-try (BF_10_ = 0.23 = 1/4.35) and anecdotal evidence against the model with only the main effect of *order* (BF_10_ = 0.67 = 1/1.49) compared to the null model. In addition, adding the interaction did not improve the models (Fig. 5, right panel).

#### Perceived competence (IMI – Perceived Competence)

As expected, there was decisive evidence for the model with only the main effect of *flow* on perceived competence (BF_10_ = 1.43×10^7^) and anecdotal evidence against the model with only the main effect of *order* (BF_10_ = 0.51 = 1/1.96) compared to the null model. In addition, adding the interaction did not improve the models.

#### Objective difficulty (Log_10_(KDR))

As expected, there was decisive evidence for the model with only the main effect of *flow* on log_10_(KDR) (BF_10_ = 3.48×10^20^) and weak evidence against the model with only the main effect of *order* (BF_10_ = 0.24 = 1/4.17) compared to the null model. In addition, adding the interaction did not improve the models.

#### Interest (IMI – Interest/Enjoyment)

The Bayesian repeated-measure ANOVA on the Interest subscale of the IMI indicated strong evidence for the model with the main effects of *flow, order,* and the flow*order interaction (BF_10_ = 14.93). There was also smaller but strong evidence for the model including only the main effect of *flow* compared to the null model (BF_10_ = 12.92), and there was anecdotal evidence against the model with only the main effect of *order* compared to the null model (BF_10_ = 0.40 = 1/2.5). Further post hoc analyses showed strong evidence for adding the main effect of *flow* (BF_10,incl_ = 15.72), anecdotal evidence against adding the main effect of *order* (BF_10,incl_ = 0.98 = 1/1.02), and weak evidence for adding the flow*order interaction (BF_10,incl_ = 3.06) to the null model.

#### Tension (IMI – Pressure/Tension)

The Bayesian repeated-measure ANOVA on the Pressure/Tension subscale of the IMI indicated that the model with the highest Bayes factor was the one including the main effects of *flow* and *order* (BF_10_ = 129), while adding the interaction very slightly reduced the Bayes factor (BF_10_ = 125). Further analyses showed decisive evidence for adding the main effect of *flow* (BF_10,incl_ = 103) and anecdotal evidence for adding the main effect of *order* (BF_10,incl_ = 1.77) or the flow*order interaction (BF_10,incl_ = 2.21) to the null model. Thus, participants experienced greater tension while playing the low-flow, more difficult game than the high-flow game matched to the participants’ skills.

#### Mental effort (RSME)

The Bayesian repeated-measure ANOVA on the RSME indicated decisive evidence for the model including the main effects of *flow* and *order,* and the flow*order interaction (BF_10_ = 2698). There was also smaller but strong evidence for the model including only a main effect of *flow* compared to the null model (BF_10_ = 45.90) and there was anecdotal evidence against adding the main effect of *order* to the null model (BF_10_ = 0.53 = 1/1.89). Further post hoc analyses showed that there was decisive evidence for adding the main effect of *flow* (BF_10,incl_ = 1210) and the flow*order interaction (BF_10,incl_ = 149) to the null model, and smaller but still strong evidence for adding the main effect of *order* (BF_10,incl_ = 38.71) to the null model. Analysis of the mean RSME values in each cell of the design showed that the participants who started with the low-flow game had similar mental effort scores in both conditions, while those who started with the high-flow game reported significantly higher mental effort scores in the low-flow more difficult condition than in the high-flow game condition matched to the participants’ skills.

#### Frustration

The average of the scores on the two frustration questions was used as the dependent measure quantifying frustration, with higher values reflecting higher frustration. The model with the highest Bayes factor included the main effects of *flow* and *order* on frustration (BF_10_ = 4354) compared to the null model, and interaction reduced the Bayes factor (BF_10_ = 3593). There was decisive evidence for the model with only the main effect of *flow* (BF_10_ = 1298) and anecdotal evidence for the model with only the main effect of *order* (BF_10_ = 1.02) compared to the null model. Thus, playing the low-flow game is indeed more frustrating than playing the high-flow game, and this difference was not influenced by the order of these conditions.

### Results: Physiology

**Fig. 6:**
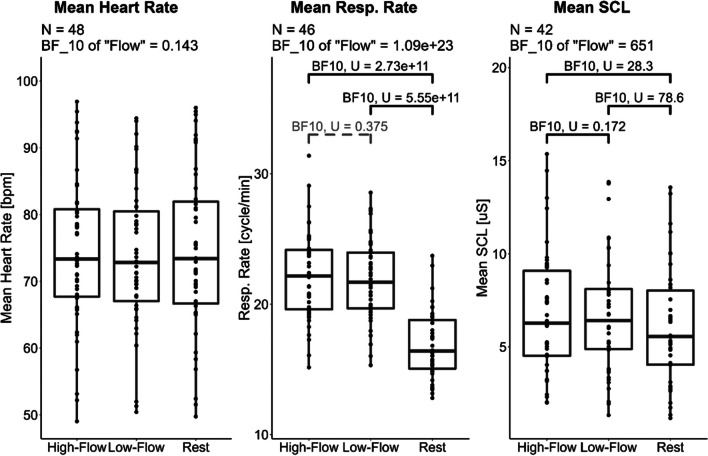
Experiment 2. Boxplots of the physiological measures indexing sympatho-vagal balance (heart rate and respiratory rate) and sympathetic activation (SCL) across our three game conditions. While high and low flow show similar physiological responses, rest shows lower respiratory rate and SCL, but no such difference was observed for heart rate. Horizontal bars represent the median and first and third quartiles. Comparisons with a Bayes factor larger than 3 are printed in black; otherwise they are printed in gray with dashed lines. While initially 49 participants were recruited for this experiment, fewer participants could be included for each measure because of motion artifacts, technical issues, or unusable data (see “Data analyses” section for more details)

**Fig. 7:**
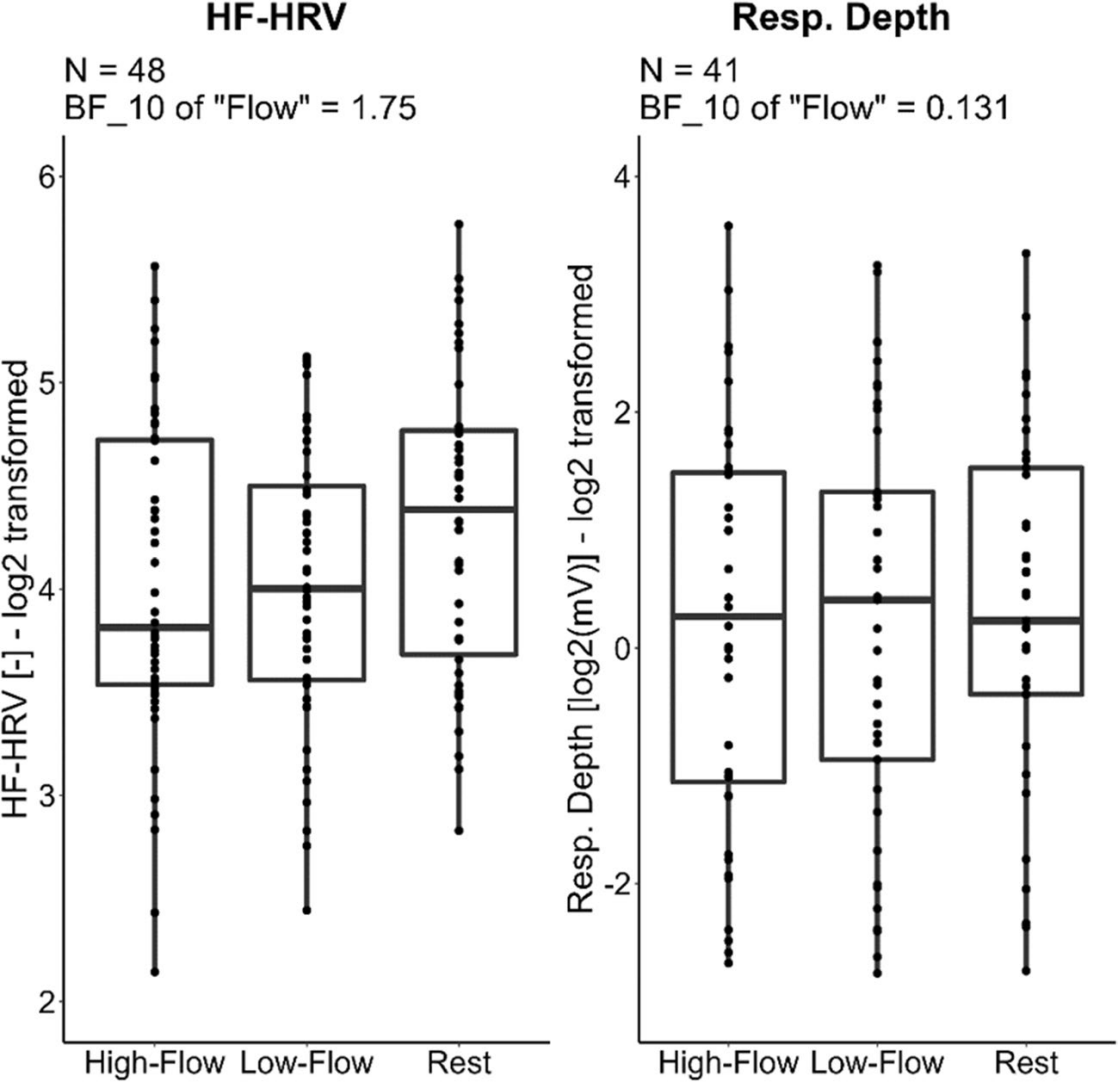
Experiment 2. Boxplots of physiological measures indexing parasympathetic activation. No difference was observed across all three conditions. Horizontal bars represent the median and first and third quartiles. While initially 49 participants were recruited for this experiment, fewer participants could be included for each measure because of motion artifacts, technical issues, or unusable data (see “Data analyses” section for more details)

We performed a Bayesian repeated-repeated measure ANOVA with *game condition* (rest, high-flow, low-flow) as within-subject factor and *order* as between-subject factor with respect to the mean heart rate and HF-HRV (cardiac measures), the mean respiratory rate and respiratory depth (respiratory measures), and the mean SCL (electrodermal activity measure). Descriptive statistics and statistical tests for all physiological measures are given in Table 4. Data for the physiological measures indexing sympatho-vagal balance (mean heart rate, respiratory rate) and sympathetic activity (SCL) are reported in Fig. 6. Data for the measures indexing parasympathetic activation (HF-HRV and respiratory depth) are reported in Fig. 7.

**Table 4:** Experiment 2. Summary table of the physiological measures during the three game conditions—rest, high-flow, and low-flow. Values in parentheses are standard deviations. A total of 48 participants were included for the cardiac measures, 46 for the respiratory rate, 41 for respiratory depth, and 42 for the skin conductance. * Denotes values where rest differs from values in the other two conditions with a level of evidence (BF_10_) higher than 10. All other comparisons had either inconclusive levels of evidence or evidence for the null hypothesis. The evidence for an effect of game condition is the Bayes factor of the model containing only the main effect of flow against the null model

	High flow	Low flow	Rest	Evidence for an effect of *game condition*
Mean heart rate [bpm]	74.6 (11.5)	74.2 (11.4)	75 (12.5)	BF_10_ = 0.14 = 1/7.14
HF-HRV [-]	4.02 (0.785)	4.02 (0.679)	4.3 (0.831)	BF_10_ = 1.75
Resp. rate [cycle/min]	22.1 (3.31)	21.8 (3.04)	16.8 (2.69)*	BF_10_ = 1.09×10^23^
Resp. depth [log_2_(mV)]	0.11 (1.74)	0.25 (1.73)	0.27 (1.56)	BF_10_ = 0.13 = 1/7.69
Mean SCL [μS]	6.8 (3.29)	6.83 (3.29)	6.05 (3.11)*	BF_10_ = 651

#### Cardiac measures (ECG)

##### Heart rate

There was weak evidence for the model with only the main effect of *game condition* on heart rate (BF_10_ = 0.14 = 1/7.14), anecdotal evidence against the model with only the main effect of *order* (BF_10_ = 0.64 = 1/1.56), and anecdotal evidence for the model with the two main effects and their interaction (BF_10_ = 1.30) compared to the null model. Accordingly, no further post hoc analyses were carried out.

##### High-frequency heart rate variability

There was anecdotal evidence for the model with only the main effect of *game condition* on HF-HRV (BF_10_ = 1.75) and weak evidence against the model with only the main effect of *order* (BF_10_ = 0.30 = 1/3.33) compared to the null model. In addition, adding the interaction did not improve the models. Accordingly, no further post hoc analyses were carried out.

#### Respiratory measures

##### Respiratory rate

There was decisive evidence for the model with only the main effect of *game condition* on the mean respiratory rate (BF_10_ = 1.09×10^23^) and weak evidence against the model with only the main effect of *order* (BF_10_ = 0.27 = 1/3.70) compared to the null model. In addition, adding the interaction did not improve the models. Post hoc tests (Bayesian paired *t*-tests) showed decisive evidence for a mean respiratory rate difference between rest and high flow (BF_10,U_ = 2.73×10^11^) and between rest and low flow (BF_10,U_ = 5.55×10^11^). There was also anecdotal evidence against a difference between mean respiratory rate in high flow and low flow (BF_10,U_ = 0.375 = 1/2.67).

##### Respiratory depth

There was weak evidence for the model with only the main effect of *game condition* on respiratory depth (BF_10_ = 0.13 = 1/7.69) and decisive evidence against adding the main effect of *order* (BF_10_ = 173) compared to the null model. In addition, adding the interaction did not improve the models. The main effect of *order* was characterized by participants who started with the high-flow condition having lower respiratory depth in all conditions than those who started with the low-flow condition.

#### Skin conductance level

The model with the highest Bayes factor included the main effects of *game condition* and *order* on the mean SCL (BF_10_ = 1317) compared to the null model, and interaction reduced the Bayes factor (BF_10_ = 651). There was decisive evidence for the model with only the main effect of *game condition* (BF_10_ = 370) and anecdotal evidence for the model with only the main effect of *order* (BF_10_ = 1.74) compared to the null model. Post hoc tests (Bayesian paired *t*-tests) showed strong evidence for a mean SCL difference between rest and high flow (BF_10,U_ = 28.3) and rest and low flow (BF_10,U_ = 78.6). There was also weak evidence against a difference between mean SCL in high flow and low flow (BF_10,U_ = 0.17 = 1/5.81).

### Discussion: Experiment 2

The goal of Experiment 2 was to replicate the main results of E1 with a larger sample and extend it to better understand different aspects of effort as well as possible associated physiological states for each condition.

Regarding differences in flow state, a similar effect size as in E1 was found, with a mean difference of 0.41 (*SD* 0.33) between our high- and low-flow conditions, corresponding to an effect size of Cohen’s *d* = 1.23 (95% CI [0.86, 1.60]). Importantly, in this experiment there was evidence against a difference in best-try between our condition with a Bayes factor of 4.35 for the null hypothesis, suggesting that we were indeed able to equalize the degree to which participants tried their best in the face of significantly different flow states.

Experiment 2 introduced the RSME scale to assess the mental effort participants reported to be required to play across flow conditions. RSME in the low-flow, more difficult condition was significantly higher than in the high-flow condition, with the difference between the conditions in the range of what is reported in previous studies using the RSME to contrast two conditions differing in mental effort (Harris et al., 2017; Rakauskas et al., 2004; Williams et al., 2002; Wilson et al., 2006). This was the case in the face of rather matched best-try (measured by the Effort/Importance subscale of the IMI) across conditions. Thus, participants reported trying their best equally across flow conditions, while rating the low-flow difficult game as requiring more effort to play well.

Regarding the other IMI variables (interest and tension) as well as frustration, numerical values indicated high interest in the face of moderate tension and frustration overall. This is in line with our expectation that participants would stay on task in both flow conditions, avoiding at low flow too much tension and frustration which can be associated with participants giving up. In addition, correlations between FSS score and frustration were low (and nonsignificant) in both conditions (*r*(high flow) = −0.18, *r*(low flow) = −0.13), suggesting that our game manipulation differentially impacted flow and frustration. Here we note that these results are unlike those reported by Tordet et al. (2021), who found that greater flow feelings were accompanied by greater frustration in the only study to our knowledge that directly investigated the link between flow and frustration. The results from Tordet et al. (2021) are in line with the idea that frustration may be an integral part of the flow experience (Csikszentmihalyi, 1990). However, unlike in our experiment, Tordet et al. (2021) measured frustration in a low-flow easy condition in addition to a matched high-flow condition and a low-flow difficult condition. While it is possible that greater frustration is associated with greater flow between an easy low-flow versus high-flow condition, it is less likely that greater frustration arising between a high-flow condition and an over-challenging low-flow condition (as was the case in our experiment) is associated with greater flow. Note that this distinction cannot be drawn from the results from Tordet et al. (2021), as they averaged frustration and flow over their three conditions (so only one value was reported for each of these constructs), nor can it be inferred from our study, as it was not equipped to answer this question. Thus, further studies may be warranted to characterize the flow-eliciting effect of frustration.

A main effect of *flow* condition on interest was observed, whereby participants reported higher interest in the high-flow than in the low-flow condition. Yet, reassuringly, the value for interest in low flow (4.8 out of 7) remained in the higher range of values reported in the literature. For example, participants reported a mean interest value of 5.5 in a rehabilitation video game (Mihelj et al., 2012), and aerobics class attendees reported average interest of 5.6 (Markland & Hardy, 1997). On the other hand, when playing an educational video game about Dutch proverbs, participants reported mean interest of 2.8 (Vos et al., 2011), when playing educational games about electrical engineering, participants reported mean interest of 2.5 (de Lima et al., 2015), and when performing a computerized psychophysics task, they reported mean interest of 2.3 (Deci et al., 1994). Thus, while participants were not as interested in the low-flow game as in the high-flow one, they were still indicating a relatively high level of interest in both conditions. Mirroring the results for interest, participants reported greater tension and frustration in the low-flow compared to the high-flow game. Importantly, values for both tension (3.2 out of 7 in high flow, and 3.8 in low flow) and frustration (2.3 out of 5 in high flow and 3.2 in low flow) remained at rather low levels. For example, in a study where participants played a video game in boredom, flow, and a frustration condition, Sharek and Wiebe (2014) reported levels of frustration in their “flow” condition at levels similar to those in our low-flow condition, and levels in our high-flow condition matched those in their boredom (easy) condition. Regarding tension, Mihelj et al. (2012) reported levels of 2.0 out of 7.0 while playing a rehabilitation video game. On the other hand, participants in an aerobics class reported a tension level of 5.4 during their class (Deci et al., 1994). These results suggest that, while the low-flow game was more frustrating and elicited more tension than the high-flow one, both of these constructs were kept at moderately low levels during the low-flow game, in line with participants remaining on task.

The second goal of this study was to investigate possible physiological differences between our flow conditions. The results from the electrodermal recording showed that sympathetic activation was similar between our flow conditions, and significantly higher than rest. Regarding parasympathetic activation, HF-HRV and respiratory depth showed no difference between the high-flow and low-flow games or with rest. These similar sympathetic and parasympathetic activations in the two flow conditions are further confirmed by the similar levels of sympatho-vagal balance observed in both conditions using heart rate and respiratory rate. These results are similar to those of Harmat et al. (2015), where the authors did not observe any difference in heart rate or HF-HRV between rest and their experimental conditions or between experimental conditions. In addition, Keller et al. (2011) did not observe any difference between rest and either of their hard and easy conditions, and de Sampaio Barros et al. (2018) did not observe any differences in HRV between their high-flow condition and their low-flow, difficult condition. These results contrast with those of Harris et al. (2017), who observed decreased HF-HRV in a high-flow condition compared to low-flow conditions that were easier or harder, and those of Tozman et al. (2015), who observed intermediate levels of HF-HRV in their high-flow condition compared to their low-flow, easy (higher HF-HRV) and hard (lower HF-HRV) conditions. Interestingly, in the studies that did not report differences in HF-HRV, the challenge of the task in the “flow” condition was matched to the skills of the participant, while in the studies that did report such differences, the challenge of the task was simply set at an arbitrary intermediate level. By not tailoring the difficulty level to each participant, the latter may have resulted in low-flow conditions either so boring or so frustrating that, as hypothesized by motivational intensity theory (Richter et al., 2016), the participants gave up. Thus, a possible explanation for the discrepancy in these results is that in the absence of a matching procedure, peripheral physiological measures may not speak to differences in flow but rather to differences in on-task/off-task behaviors.

Given the interest in investigating the neural correlates of flow using brain imaging techniques and our aim of developing a procedure that allows real-time monitoring of the neural bases of flow, Experiment 3 ports our methodology within the environment of an MRI scanner to investigate whether our results on the FSS and Best-Try scale would hold in an arguably noisier environment.

## Experiment 3

Experiments 1 and 2 presented a procedure to systematically induce both low- and high-flow conditions, all the while ensuring participants tried their best equally in both conditions. Generating such experimental conditions in a reliable fashion is an important step toward investigating flow in a controlled way, that is unconfounded by off-task behaviors that either boredom or high frustration may trigger. In Experiment 3, we ask whether these first results can be replicated within the noisy and confined MRI environment where participants play video games in a supine position, imposing unusual constraints on their movements.

As in E2, the physiological correlates of the induced high- and low-flow states were monitored in an effort to clarify the robustness of the null effects between high and low flow in terms of sympathetic and parasympathetic markers. Experiment 3 also addresses a possible limitation of the E2 design regarding cardiac measures at rest. Indeed, the rest period was quite close in time to the evaluation game, and anticipation at the prospect of game play in subsequent conditions may not have given enough time for cardiac activity to return to a true resting baseline level (Pulopulos et al., 2018). To address this issue, Experiment 3 uses a longer rest period (12 minutes instead of 5) that is separated in time from gaming periods by at least a couple of minutes.

### Materials and methods

#### Participants

Thirty healthy individuals participated in this study, 21 men and 9 women between 18 and 32 years of age (mean age = 21.8, SD = 2.8 years), recruited on the same criteria as E1 and E2 except for one change: Due to a shortage of potential participants, participants’ shooter play hours per week was not used as an inclusion criterion. However, we kept the in-lab *UT2004* screening game inclusion criterion. We posited that participants who were able to spontaneously perform this well on *UT2004* had enough gaming experience to be enrolled in our study. The results from E3 show that, using only this latter criterion, flow induction was similar to that in E1 and E2. Participants with a KDR between 0.5 and 4 in their second 10-minute game were invited to participate in the study; two participants played only one 10-minute game, in which case we used the KDR for this game. All participants reported being comfortable with shooter games.

Participants were given information about the experiment and were asked to provide informed consent in accordance with the Declaration of Helsinki, under a protocol approved by the ethics commission of the Faculty of Psychology and Educational Sciences of the University of Geneva, and were paid for their participation.

#### Design

Participants were lying down as behavioral, physiological, and MRI measures were recorded. Here we only report on the behavioral and physiological data; the MRI measures were exploratory. Among others, our design included three “conditions”: a resting state period followed by two periods where the participants played the same game as in E1 and E2 (*UT2004*) at a high or low level of flow as in E1 and E2. The difficulty of the game for the low-flow and high-flow conditions was set using the same protocol as in E1 and E2. In particular, participants completed both the initial screening and evaluation game play as in E1 and E2 (i.e., in a normal sitting position outside the scanner). In the main experiment, the order of the low-flow and high-flow conditions was counterbalanced across subjects, and self-report questionnaires were administered immediately after each game play period, with the instruction to answer these questionnaires based on their experience during the *last game only*. Finally, participants performed a 10-minute sustained attention and inhibition task, after which the same questionnaires were administered. Descriptions of the task and of the results from the physiological and behavioral measures linked to the task are reported in the supplementary materials (SM-06) but will not be discussed further.

#### Materials and apparatus

##### Questionnaires and skill measures

**Flow (FSS), motivation measures (best-try, interest, tension, perceived competence), RSME, and frustration.** Same as in Experiment 2.

**Flow proneness and emotionality questions.** Two exploratory questionnaires, one about flow proneness—the Swedish Flow Proneness Questionnaire (Ullén et al., 2012)—and another about emotional status, were collected. As for the other questionnaires, participants were presented each item with its French translation and its original English version in italicized font just underneath. These questionnaires were outside the scope of the present study and thus will not be discussed further. Results for these questionnaires can be found in the supplementary materials (SM-05).

##### Physiological measures

Cardiac, electrodermal, and respiratory measures were all recorded using the Biopac MP150 system at 2000 Hz and AcqKnowledge 4.4 software. In addition, all physiological signals were converted into an optical signal in the scanner room and underwent electro-optical conversion outside the scanner room to be processed by the Biopac MP150 module.

**Heart rate and high-frequency heart rate variability.** Due to technical constraints imposed by the MRI environment, we could not use an ECG to acquire cardiac measurements, and photo-plethysmography (PPG) was used instead. A review of the literature comparing cardiac measures such as heart rate and frequency-domain measures of heart rate variability has shown that measures from PPG can be used as surrogates for measures derived from an ECG with sufficient reliability in contexts that do not involve exercise or participants with cardiovascular diseases, i.e., such as in the present study (Schäfer & Vagedes, 2013). For these reasons and for the sake of clarity, we will denote cardiac measures derived from PPG with the same terms as those derived from ECG.

The plethysmograph was placed on one of the participant’s big toes and connected to the Biopac PPG100 module. As in E2, heart rate was measured as the inverse of the mean duration between two peaks in the pulse plethysmogram, and HF-HRV was measured as the power of the high-frequency band (0.15–0.4 Hz) normalized by the sum of the powers of the very low-frequency (0–0.04 Hz), low-frequency (0.04–0.15 Hz), and high-frequency (0.15–0.4 Hz) bands as computed in Kubios analysis software (Tarvainen et al., 2014, version 2.3). As in E2, heart rate and HF-HRV were used as proxies for the sympatho-vagal balance and the parasympathetic activity, respectively.

**Respiratory rate and depth.** Respiratory data were acquired using the MRI-compatible Biopac respiratory belt strapped around the lower part of the ribcage of the participant and connected to a Biopac RSP100C module. As with PPG, the signal was first converted into an optical signal and underwent electro-optical conversion outside the scanner room to be processed by the Biopac RSP100C module. The belt was fastened so that it did not impair breathing but was sufficiently tight that it would not slide down during the recording. Respiratory rate was measured as the inverse of the period between two peaks in the respiratory trace, and respiratory depth as the mean amplitude of each peak. Respiratory depth was used as a proxy for parasympathetic activation, and respiratory rate as a proxy for sympatho-vagal balance (Lumma et al., 2015; Wientjes, 1992).

**Skin conductance level.** SCL was recorded using two Ag/AgCl single-use electrodes filled with saline electrode gel connected to a Biopac EDA100C MRI module. Since participants were using both hands to play the game, and to avoid additional noise from the scanner, the SCL signal was recorded from the foot sole as described in van Dooren et al. (2012), where good correlation with SCL acquired on the hands was found. Gel and set up on the foot sole followed the directions in van Dooren et al. (2012), i.e., position of the electrodes and cleaning of the foot sole before recording. The electrodes were placed on the foot without the pulse plethysmograph and connected to the Biopac module through an optical fiber. We used mean SCL as a proxy for sympathetic activation (Boucsein, 2012).

##### Gaming equipment

All visual stimuli were presented on a 23″, 1920×1080 MRI-compatible screen (Cambridge Research System, Rochester, Kent, UK). To play in the scanner, the participants were provided with custom-made MRI-compatible mouse and keyboard that behaved similarly to commonly found equivalent hardware outside the scanner. The keyboard was put on a custom-designed mini-workspace consisting of a main surface with the computer mouse set on a smaller surface above the keyboard. The total apparatus rested on foam cushions placed on the waist and thighs of the participant. The smaller surface was designed to cover only the right half of the keyboard, such that the keys necessary to play the FPS were still accessible (namely the space bar and the WASD keys). A guiding piece of tape was put on the W key so that the participants would be able to put their hand back in place in case they were to move it and “lose” the position of the WASD keys. The keyboard and mouse were connected to an electrical-to-optical signal converter to reduce interference from the magnetic field as much as possible and converted back to electrical signals outside the scanner room. Foam was put below the participants’ elbows for comfort. The position of the custom-designed workspace was arranged so that the keyboard and mouse were at arm’s length when lying down and that maintaining this position for 90 minutes was comfortable. When asked, no subject expressed feeling discomfort during play.

#### Procedure

**Fig. 8:**
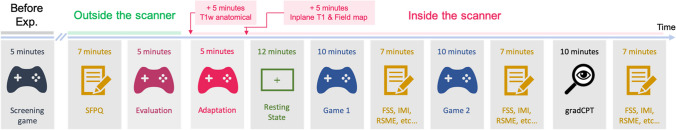
MRI study protocol. The protocol lasted 2.5 hours. A half-hour was dedicated to welcoming the participant and titrating game play level outside the scanner. Note that structural MRI images were recorded before the MR-adaptation video game play session and in-plane-T1 and field maps immediately after that game play

Participants were first given information about the experiment and filled out the consent form and the MRI safety questionnaire. They then completed the flow proneness questionnaire (not discussed further), and completed the exact same 5-minute evaluation game as in E1 and E2. Game levels for the high- and low-flow conditions were then determined using the same procedure as in E1 and E2 (Fig. 1). This included using their KDR during a 10-minute screening game that occurred before the experiment (screening game in Fig. 8). Next, participants were escorted to the scanner room. If the participant was wearing glasses, they were also provided with MRI-compatible goggles matching their usual correction. They were then set up with the physiological apparatus and given the instructions for the in-scanner play and MRI procedure. Finally, they were comfortably installed in the magnet and provided with the MRI-compatible keyboard and mouse.

In the scanner, the participants successively completed five conditions (Fig. 8): a 5-minute MR-adaptation game, a rest period, the high-flow and low-flow game sessions counterbalanced in order across subjects, and finally a sustained attention and inhibition task that will not be discussed further. After each gaming session (high-flow and low-flow), participants were asked to fill out the FSS, the IMI subscales (measuring *best-try, interest, perceived competence, and tension*), the RSME, and the questions on frustration (and emotions—not discussed further) as described in the “Questionnaires” section.

- **MR-adaptation game**: The goal of this game was for the participant to get used to playing in the scanner, as conditions are different from playing seated in front of a desk. Participants played for 5 minutes, with the same settings as in the evaluation game played outside of the scanner, except that the map was changed to *Rankin.* The bots’ level was set at the flow level obtained from the evaluation game. If the participant’s KDR was lower than 0.5 at the end of the MR-adaptation game, the high-flow and low-flow levels were lowered by 1 for the rest of the experiment. Images were recorded during this MR-adaptation game to help participants get used to the noise of the scanner while playing. These recordings were not used for analysis.
- **Resting state**: Participants were asked to fixate on a white cross (size: 1.3° visual angle, thickness: 0.18°) in the middle of a black screen for 12 minutes and were told that they could think about whatever they wanted. They were explicitly instructed not to close their eyes for extended periods of time or to fall asleep. We used the eye tracker camera to monitor their eyes during this period to control whether they fell asleep. Physiological data recorded during this resting state period served as baselines for the other physiological measures. One participant started falling asleep after 9 minutes 30 seconds out of the 12 minutes of the session. Given that 9.5 minutes is sufficient time to get a good estimate of baseline values for our physiological measures, we used the values measured during these 9.5 minutes as baseline values for this participant.
- **Games (high-flow vs. low-flow):** Same as in E2, except for playing position. Participants played in a supine position with the keyboard and mouse resting on their lap, as described in the “Gaming Equipment” section.

### Data analysis

For all physiological measures, raw data were first converted into MATLAB format using the default AcqKnowledge save function. Then using EEGLab (Delorme & Makeig, 2004) functions, data were band-pass-filtered between 0.5 Hz and 5 Hz (cardiac measures), band-pass-filtered between 0.05 Hz and 1 Hz (respiratory measures), or low-pass-filtered at 1 Hz (SCL) to remove the artifacts from the fMRI scans and the slow components, following the recommendation from Biopac^®^.

#### Cardiac measures (PPG)

The MATLAB function *findpeaks* was used to identify the peaks in the filtered signal from the plethysmograph, followed by visual inspection of the raw PPG trace to either remove the peaks that were mere artifacts or add peaks that were missed. The inter-beat interval (IBI) series was then exported and analyzed using HRVAS (Ramshur, 2010), a MATLAB toolbox dedicated to HRV analyses. We report here the mean heart rate and the power of the high-frequency band (0.15–0.4 Hz, Task Force of the European Society of Cardiology and the North American Society of Pacing and Electrophysiology, 1996) of the IBI series normalized by the total power of the spectrum (HF-HRV). Out of the 30 participants, three had to be excluded because of excessive artifacts that could not be corrected, and correction was needed for 14 of them in one of the conditions (rest, high-flow, or low-flow). The number of peaks that needed correction varied from 1 to 40 (~7% of the total number of peaks). This gave us a final sample size of 27 for cardiac measures.

#### Respiratory measures

The filtered signal was first down-sampled from 2000 Hz to 50 Hz to reduce computational load. Then a similar method as the one described in E2 was used to identify the respiratory cycles. The respiratory rate was then computed as the mean of the inverse of the inter-peak intervals, and respiratory depth was measured by the average amplitude of the peaks across the session. Mean respiratory depth values were further log_2_-transformed so that they would follow a normal distribution.

Seven subjects were excluded from the analyses because of technical issues (five of them) or excessive motion (two of them) during at least one of the runs. In addition, for five subjects, we could not use the whole recording but there was enough good data to extract meaningful value. Instead of the 10 minutes for the games and 12 minutes for rest, we used between 3 minutes 20 seconds and 9 minutes 23 seconds of data. These durations are in the range of usual durations for respiratory recordings in psychophysiology (e.g., Grassmann et al., 2016). Finally, outlier values for cycle length and amplitude, defined as values more than three standard deviations from the mean, were excluded from analysis. At most 10 peaks were excluded from analysis, for an average of three peaks per condition (about 2% of the recorded peaks). This gave us a final sample of 23 for respiratory measures.

#### Skin conductance level

The signal was first down-sampled from 2000 Hz to 50 Hz to reduce computational load. Tonic and phasic components of the signal were extracted using a method similar to the one implemented in AcqKnowledge (Dawson et al., 2000): The signal was divided into a high-frequency and a low-frequency signal, corresponding to the tonic and phasic components, respectively, using a low- and a high-pass filter with a cutoff frequency of 0.05 Hz. Then the skin conductance level (SCL) was extracted as the mean value of the tonic component. Five participants exhibited no response in terms of skin conductance level and thus were excluded from the analyses, yielding a final sample size of 25 for SCL.

### Results: Behavior

As in E1 and E2, Bayesian repeated-measure ANOVAs with *flow* (*high flow* or *low flow*) as within-subject factor were carried out on all dependent variables (namely *FSS, best-try, perceived competence, KDR, interest, tension, RSME, frustration*). As above, we also coded whether participants started with a high-flow game or a low-flow game with a between-subject “*order*” factor. The same guide as for E1 and E2 on how to interpret results from these ANOVAs can be found in the supplementary materials (SM-07). Descriptive statistics for all the dependent measures in each condition are given in Table 5. We also report the results of the frequentist repeated-measure ANOVA on the FSS in the supplementary materials (SM-08) as means of comparison with the results of the Bayesian analyses. Descriptive statistics for all the dependent measures in each condition are given in Table 5.

**Table 5:** Experiment 3. Descriptive statistics of all the dependent behavioral measures in E2 (*N* = 30). The average is reported, with the standard deviation in parentheses. The evidence for an effect of flow condition is the Bayes factor of the model containing only the main effect of flow against the null model. Non-log-transformed KDR values are given for information, as they are more straightforward to interpret than the log-transformed values used in the analyses

	High flow	Low flow	Evidence for an effect of flow condition
*Predicted to differ*			
FSS	3.61 (0.43)	3.21 (0.35)	BF_10_ = 4.9×10^6^
*Predicted to be matched*			
Best-try (IMI)	5.73 (0.89)	5.43 (0.97)	BF_10_ = 1.21
*Variables predicted to differ*			
Perceived competence (IMI)	3.7 (1.40)	2.3 (1.04)	BF_10_ = 2.16×10^5^
Log_10_(KDR)	0.13 (0.24)	−0.60 (0.31)	BF_10_ = 1.36×10^13^
KDR	1.34	0.25	
*Variables predicted to remain low*			
Tension (IMI)	3.25 (1.02)	3.33 (1.33)	BF_10_ = 0.29 = 1/3.45
Interest (IMI)	4.84 (1.33)	4.48 (1.50)	BF_10_ = 3.2
Effort (RSME)	61.8 (24.9)	77.0 (25.7)	BF_10_ = 472
Frustration	2.0 (0.89)	3.0 (1.19)	BF_10_ = 1.41×10^5^

#### Flow score (FSS)

**Fig. 9:**
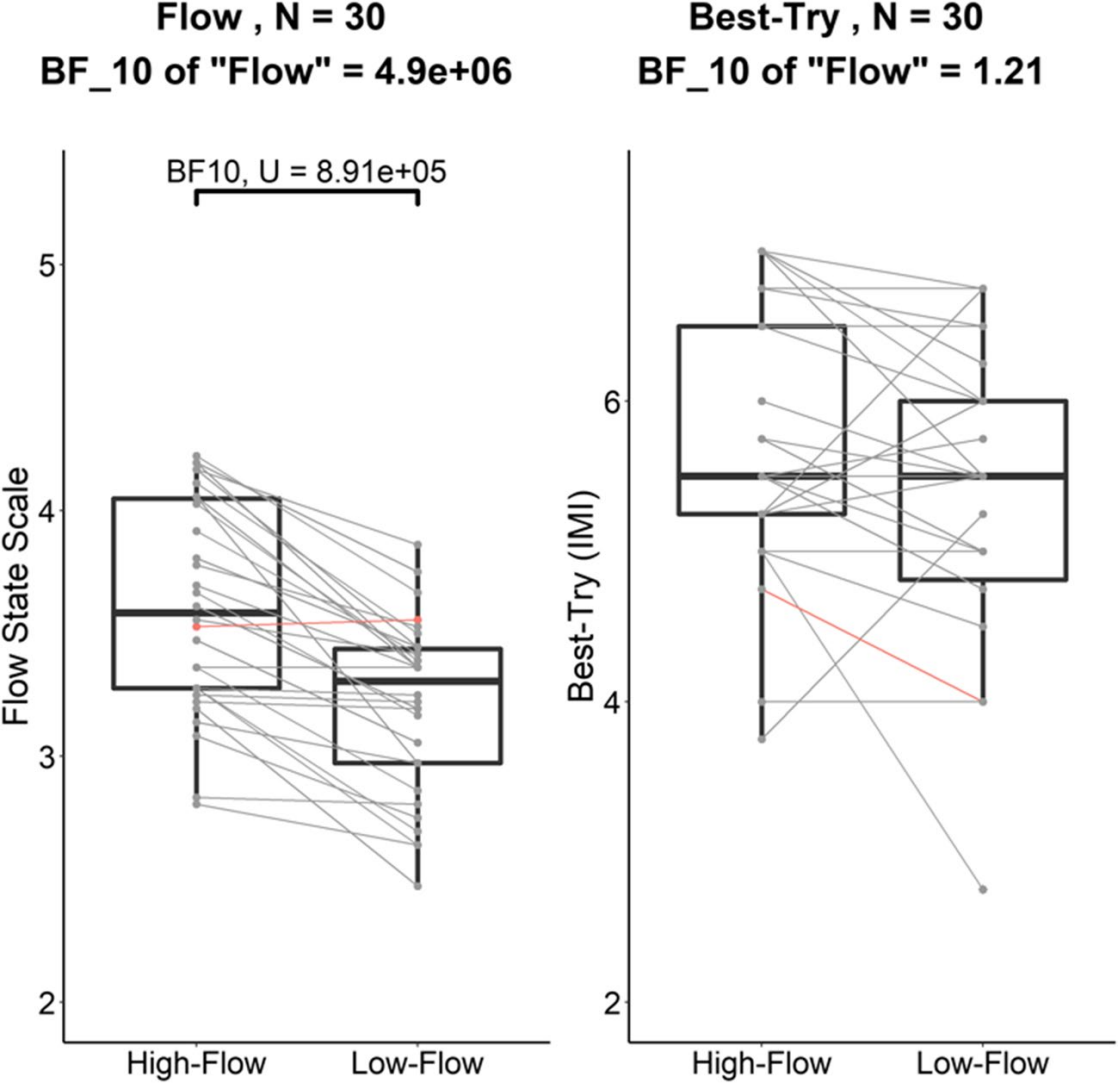
Experiment 3. Boxplots of the individual scores on the FSS (left) and best-try (right) in the high-flow and low-flow conditions with the individual scores superimposed. The color of the links on the left plot indicates whether the difference in FSS between high flow and low flow was positive (gray—as expected) or negative (red)

The Bayesian repeated-measure ANOVA on the FSS score with *flow* (high vs. low flow) as within-subject factor and *order* as a between-subject factor indicated decisive evidence for the model with only the main effect of *flow* (BF_10_ = 4.90×10^6^) compared to the null model; there was anecdotal evidence against the model with only the main effect of *order* (BF_10_ = 0.39 = 1/2.56), and adding their interaction did not improve the model (Fig. 9, left panel).

#### Best-try (IMI – Effort/Importance)

In line with best-try being matched across flow conditions, there was anecdotal evidence for the model with only the main effect of *flow* on best-try (BF_10_ = 1.21) and anecdotal evidence against the model with only the main effect of the *order* factor (BF_10_ = 0.44 = 1/2.72) compared to the null model. In addition, adding the interaction did not improve the models (Fig. 9, right panel).

#### Perceived competence (IMI – Perceived Competence)

As expected, there was decisive evidence for the model with only the main effect of *flow* on perceived competence (BF_10_ = 2.16×10^5^) and anecdotal evidence against the model with only the main effect of *order* (BF_10_ = 0.35 = 1/2.86) compared to the null model. In addition, adding their interaction did not improve the model.

#### Objective difficulty (Log_10_(KDR))

As expected, there was decisive evidence for the model with only the main effect of *flow* on Log_10_(KDR) (BF_10_ = 1.36×10^13^) and weak evidence against the model with only the main effect of *order* (BF_10_ = 0.32 = 1/3.13) compared to the null model. In addition, adding their interaction did not improve the model.

#### Interest (IMI – Interest/Enjoyment)

The Bayesian repeated-measure ANOVA on the Interest subscale of the IMI indicated weak evidence for the model with only the main effect of *flow* on interest (BF_10_ = 3.2) and anecdotal evidence against the model with only the main effect of *order* (BF_10_ = 0.55 = 1/1.81) compared to the null model. There was, however, very strong evidence in favor of the model with the interaction between these two factors (BF_Incl_ = 80). Further analyses showed that there was weak evidence against an influence of *flow* on interest when participants played the low-flow game before the high-flow game (BF_10_ = 0.17 = 1/5.88). However, there was decisive evidence supporting the hypothesis that interest was higher during the high-flow condition than the low-flow condition (BF_10_ = 129) when participants started with the high-flow game.

#### Tension (IMI – Pressure/Tension)

There was weak evidence against the model with only the main effect of *flow* on tension (BF_10_ = 0.30 = 1/3.33) and anecdotal evidence for the model with only the main effect of *order* (BF_10_ = 1.2) compared to the null model. In addition, adding their interaction did not improve the model.

#### Mental effort (RSME)

There was decisive evidence for the model with only the main effect of *flow* on the effort it took participants to play the game (BF_10_ = 472) and anecdotal evidence against the model with only the main effect of *order* (BF_10_ = 0.77 = 1/1.30) compared to the null model. In addition, adding their interaction did not improve the model.

#### Frustration

As in E2, the average of the scores on the two frustration questions was used as the dependent measure quantifying frustration, with higher values reflecting higher frustration. The model with the highest Bayes factor included the main effects of *flow* and *order* on frustration (BF_10_ = 6.22×10^5^), while adding the interaction slightly reduced the Bayes factor (BF_10_ = 4.49×10^5^). Further post hoc analyses showed decisive evidence for adding the main effect of *flow* (BF_10_ = 1.81×10^5^), anecdotal evidence for adding the main effect of *order* (BF_10_ = 2.12), and weak evidence for adding the flow*order interaction (BF_10_ = 4.95) to the null model. Thus, while playing the low-flow game is indeed more frustrating than playing the high-flow game, as expected, the difference in frustration experienced between the high-flow and low-flow games was not influenced by the order of these conditions.

### Results: Physiology

**Fig. 10:**
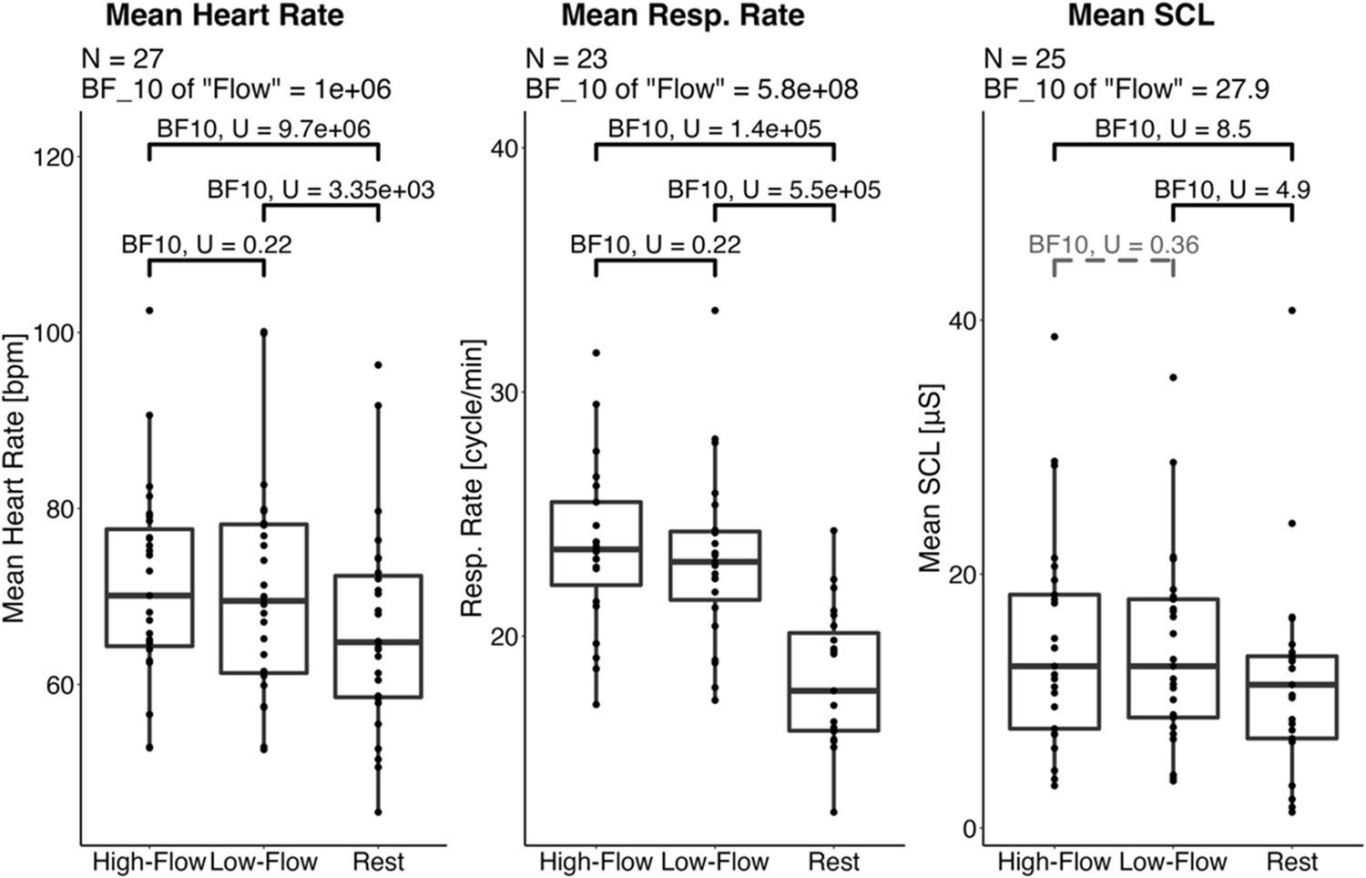
Experiment 3. Boxplots of the results for the physiological measures of sympatho-vagal balance (heart rate and respiratory rate) and sympathetic activation (SCL). While high and low flow show similar physiological responses, rest shows lower heart rate, respiratory rate, and SCL. Horizontal bars represent the median and first and third quartiles. Comparisons with a Bayes factor larger than 3 are printed in black; otherwise they are printed in gray with dashed lines. While initially 30 participants were recruited for this experiment, fewer participants could be included for each measure because of motion artifacts, technical issues, or unusable data (see “Data analyses” section for more details)

**Fig. 11:**
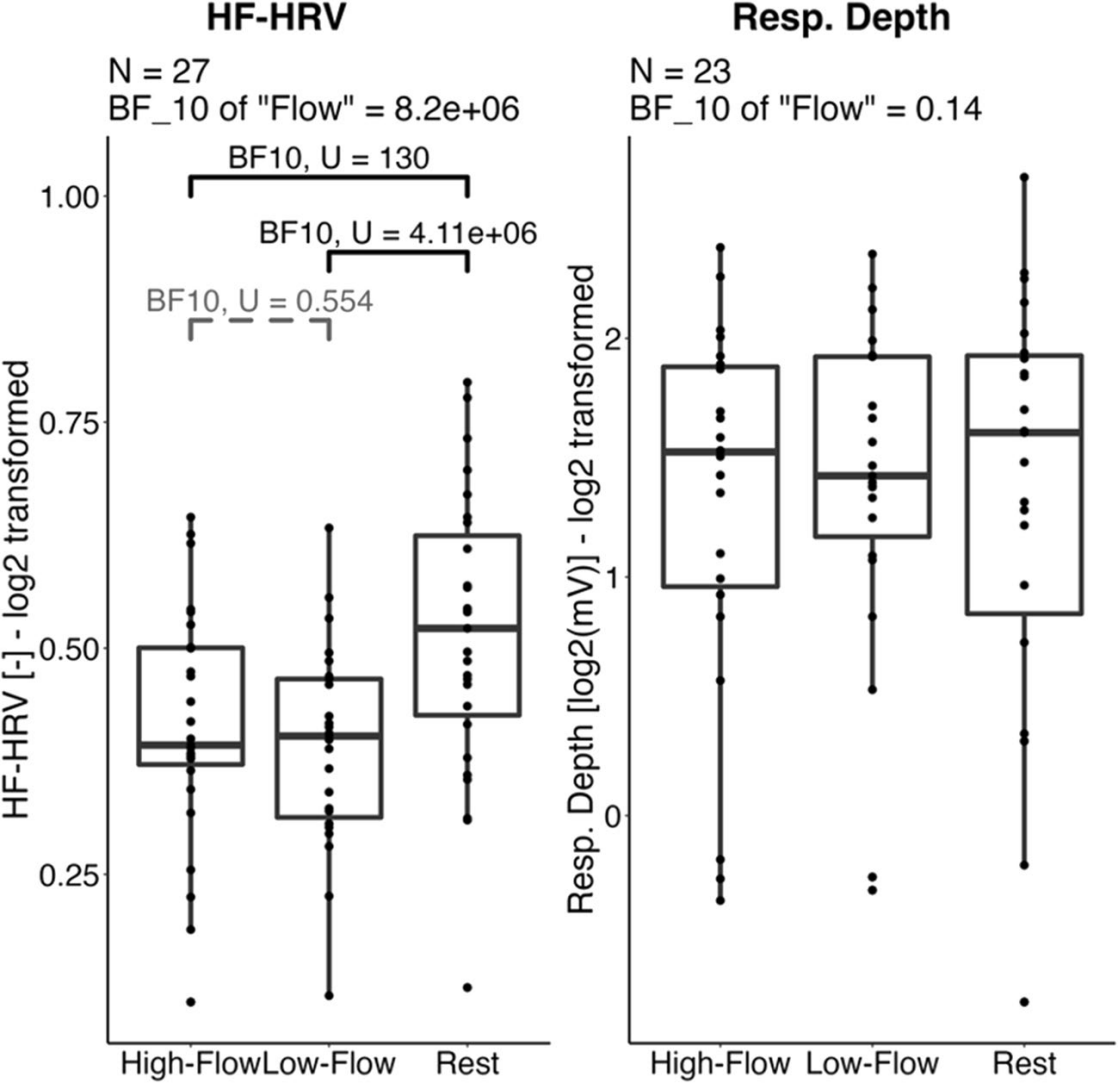
Experiment 3. Boxplots of the results for the physiological measures of parasympathetic activation. Horizontal bars represent the median and first and third quartiles. Comparisons with a Bayes factor larger than 3 are printed in black; otherwise they are printed in gray with dashed lines. While initially 30 participants were recruited for this experiment, fewer participants could be included for each measure because of motion artifacts, technical issues, or unusable data (see “Data analyses” section for more details)

We performed a Bayesian repeated-repeated measure ANOVA with *game condition* (rest, high-flow, low-flow) as within-subject factor and *order* as between-subject factor on the mean heart rate and HF-HRV (cardiac measure), the mean respiratory rate and respiratory depth (respiratory measures), and the mean SCL (electrodermal activity measure). Descriptive statistics and statistical results for all physiological measures are given in Table 6. Data for the physiological measures indexing sympatho-vagal balance (mean heart rate, respiratory rate) and sympathetic activity (SCL) are reported in Fig. 10. Data for the measures indexing parasympathetic activation (HF-HRV and respiratory depth) are reported in Fig. 11.

**Table 6:** Experiment 3. Summary table of the physiological measures during the rest, high-flow, and low-flow conditions. Values in parentheses are standard deviations. A total of 27 participants were included for the cardiac measures, 23 for the respiratory measures, and 25 for the skin conductance. * Denotes values different at rest different from values in the experimental conditions with a level of evidence (BF_10_) higher than 10. All other comparisons had either inconclusive levels of evidence, or evidence for the null hypothesis. The evidence for an effect of game condition is the Bayes factor of the model containing only the main effect of flow against the null model

	High flow		Low flow	Rest	Evidence for an effect of *game condition*
Mean heart rate [bpm]	71.1 (11.36)	70.8 (12.03)		66.2 (11.68)*	BF_10_ = 1.0×10^6^
HF-HRV [-]	0.42 (0.13)	0.39 (0.11)		0.52 (0.16)*	BF_10_ = 8.2×10^6^
Resp. rate [cycle/min]	23.67 (3.37)	23.17 (3.55)		18.27 (2.79)*	BF_10_ = 5.8×10^8^
Resp. depth [log_2_(mV)]	1.31 (0.77)	1.39 (0.69)		1.34 (0.87)	BF_10_ = 0.14 = 1/7.14
Mean SCL [μS]	14.5 (8.03)	14.0 (7.63)		11.6 (8.03)*	BF_10_ = 27.9

#### Cardiac measures (PPG)

##### Heart rate

There was decisive evidence for the model with only the main effect of *game condition* on heart rate (BF_10_ = 1.0×10^6^) and anecdotal evidence against the model with only the main effect of *order* (BF_10_ = 0.89 = 1/1.12) compared to the null model. In addition, adding the interaction did not improve the models. Post hoc tests (Bayesian paired *t*-tests) showed decisive evidence for a mean heart rate difference between rest and high flow (BF_10,U_ = 9.7×10^6^) and between rest and low flow (BF_10,U_ = 3347). There was weak evidence against a mean heart rate difference between high flow and low flow (BF_10,U_ = 0.22 = 1/4.55).

##### High-frequency heart rate variability

There was decisive evidence for the model with only the main effect of *game condition* on HF-HRV (BF_10_ = 8.2×10^6^) and anecdotal evidence against the model with only the main effect of *order* (BF_10_ = 0.67 = 1/1.49) compared to the null model. In addition, adding the interaction did not improve the models. Post hoc tests (Bayesian paired *t*-tests) showed that there was decisive evidence for an HF-HRV difference between rest and high flow (BF_10,U_ = 130) and between rest and low flow (BF_10,U_ = 4.1×10^6^). There was anecdotal evidence against a difference in HF-HRV in high flow and low flow (BF_10,U_ = 0.55 = 1/1.82).

#### Respiratory measures

##### Respiratory rate

There was decisive evidence for the model with only the main effect of *game condition* on the mean respiratory rate (BF_10_ = 5.8×10^8^) and anecdotal evidence against the model with only the main effect *order* (BF_10_ = 0.81 = 1/1.23) compared to the null model. In addition, adding the interaction did not improve the models. Post hoc tests (Bayesian paired *t*-tests) showed decisive evidence for a mean respiratory rate difference between rest and high flow (BF_10,U_ = 1.4×10^5^) and between rest and low flow (BF_10,U_ = 5.5×10^5^). There was also weak evidence against a difference between mean RR in high flow and low flow (BF_10,U_ = 0.22 = 1/4.55).

##### Respiratory depth

There was weak evidence against the model with only the main effect of *game condition* on respiratory depth (BF_10_ = 0.14 = 1/7.14), and anecdotal evidence against the model with only the main effect *order* (BF_10_ = 0.64 = 1/1.56) and the model with the two main effects and the interaction (BF_10_ = 0.88 = 1/1.14) compared to the null model. Accordingly, no further post hoc analyses were carried out.

#### Skin conductance level

There was strong evidence for the model with only the main effect of *game condition* on the mean SCL (BF_10_ = 27.9) and anecdotal evidence against the model with only the main effect *order* (BF_10_ = 0.65 = 1/1.55) compared to the null model. In addition, adding the interaction did not improve the models. Post hoc tests (Bayesian paired t-tests) showed weak evidence for a mean SCL difference between rest and high flow (BF_10,U_ = 8.5) and between rest and low flow (BF_10,U_ = 4.9). There was also anecdotal evidence against a difference between mean SCL in high flow and low flow (BF_10,U_ = 0.36 = 1/2.77).

### Discussion: Experiment 3

Experiment 3 reproduced the paradigm from E2 in the context of an MRI scanner where participants played their assigned video game sessions in supine position. This experiment adds to a growing body of studies using a complex gaming environment in the scanner (Huskey et al., 2018; Klasen et al., 2012) showing that it is possible to induce flow using video games even in the noisy magnet environment.

As in E1 and E2, the manipulation of flow held with an average difference in FSS of 0.40 (*SD* 0.28) between our high- and low-flow conditions (Cohen’s *d* = 1.42, 95% CI [0.90, 1.92]). In addition, the impact of the flow manipulation was rather robust, with all but one participant reporting higher flow in the low-flow than in the high-flow condition when measured by the FSS. These significant differences in flow ratings between conditions coexisted with matched and rather high level of best-try across conditions.

The other IMI scales and the RSME showed similar results as in E1 and E2. Participants rated the mental effort needed to complete the low-flow game higher than that required to complete the high-flow game as measured with RSME, and the high-flow game was of slightly greater interest. Order effects seen in E2 for the RSME appear now in E3 for interest, whereby the low-flow condition is rated as of greater RSME/lower interest when preceded by the high-flow condition, but not for the reverse order. Given these order effects, it may be advisable in future studies to either start with the low-flow condition and only introduce the high-flow condition second, or introduce intermediate activities to reduce such interference effects. Regarding tension and frustration, tension levels were lower in the low-flow condition in this experiment than in E2, so much so that there was no difference in tension levels between our two conditions. Frustration showed similar values as in E2, and we replicated the negative correlation between FSS scores and reported frustration (*r*(high flow) = −0.33, *r*(low flow) = −0.37). While stronger than in E2, these correlations were not significant for this sample size. Altogether, these results confirm that we were able to separate flow and frustration, and that the low-flow condition did not generate excessive frustration or tension, even in a difficult environment such as an MRI scanner.

Finally, turning to the physiological data and the hypothesis that the flow state is characterized by the coactivation of the sympathetic and parasympathetic branches of the autonomic nervous system, our results are again mixed (although convergent with the results from E2). Being on task—whether playing at low or high flow—led to sympathetic activation compared to rest. Accordingly, an increase in mean heart rate, respiratory rate, and SCL was observed in the rest condition as compared to both the low- and the high-flow conditions. Comparing between just low and high flow suggested comparable SCL and thus sympathetic activation across these two on-task conditions. This is in line with the measures of heart rate and respiratory rate, two measures of sympatho-vagal balance, which also showed greater activation in the rest condition than in the game conditions (higher heart rate and respiratory rate). These measures were also similar in both our conditions, which would suggest similar sympathetic and parasympathetic activation. These results are in line with those of Harmat et al. (2015), but contrast with those of de Sampaio Barros et al. (2018), who found higher heart rate in their difficult low-flow condition than in their medium high-flow condition, and those of Harris et al. (2017), who conversely found higher heart rate in their intermediate condition than in their difficult one.

To further address whether the parasympathetic nervous system was co-activated with the sympathetic nervous system in high flow but not in low flow, HF-HRV and respiratory depth were considered. As in E2, respiratory depth lacked sensitivity, not even distinguishing between gaming conditions and rest across both experiments, calling into question its sensitivity as a measure of parasympathetic activation in our paradigm. This lack of sensitivity is in line with a review of its use in relation to cognitive load. Grassmann et al. (2016) report that, from 24 studies which considered respiratory depth, only three of them document a difference in respiratory depth between rest and task conditions. Our other measure of parasympathetic activity, HF-HRV, showed a similar pattern as SCL, with HF-HRV being higher in the rest condition than in the high- and low-flow conditions, but it did not differ between these two. The lack of difference between high and low flow is in line with a rather mixed body of literature. In addition, even when using an ECG to measure HRV, de Sampaio Barros et al. (2018) and Harmat et al. (2015) failed to observe differences in HRV between their high-flow and hard, low-flow conditions, while Tozman et al. (2015) reported higher HRV and Harris et al. (2017) lower HRV in their high-flow condition than in their hard, low-flow condition.

## Discussion

To experimentally manipulate and characterize the state of flow, we designed a systematic method to tailor video game play difficulty to the participant’s skills inside *Unreal Tournament* 2004. We designed two individualized experimental conditions for each participant: (i) a high-flow condition in which the skill-to-challenge level is individually titrated to be balanced, and (ii) from this level, a more difficult game play condition is implemented through a systematic increase in game difficulty, yielding a low-flow, more difficult condition. This procedure requires two evaluation games of 10 minutes (“screening game” in Fig. 1) and 5 minutes (“evaluation game” in Fig. 1) to titrate difficulty before deploying the main experimental conditions to manipulate challenge at different levels and impact flow. This method yielded robust intra-individual differences in flow as measured by one of the most standard questionnaires in the field—the FSS (Jackson & Marsh, 1996). Over three experiments, the effect size for the differences in flow scores across our high- and low-flow conditions was of Cohen’s *d* 1.36, 1.23, and 1.42, with an overall effect size of 1.31 (95% CI [1.03, 1.59]) when all the participants were pooled together, indicating a large effect. In addition, the mean flow values reported by participants in all three studies (range [3.61; 3.81] for the high-flow conditions, and [3.21; 3.33] for the low-flow conditions) are in the range of FSS values reported in a study where participants played a similar video game (mean FSS of 3.76; Kivikangas, 2006). Among the 91 participants tested, only four reported lower flow scores in the high-flow than the low-flow condition. These robust flow differences were found even when the game was played without any soundtrack, as well as when participants played, with no sound, in supine position in the tube of an MRI scanner.

Importantly, the novel procedure ensured that participants remained on task, trying their best in both flow conditions. This is key, as the flow state is a special case of being on task where “*people are so involved in an activity that nothing else seems to matter; the experience itself is so enjoyable that people will do it even at great cost, for the sheer sake of doing it*” (Csikszentmihalyi, 2020). Thus, any baseline task for studying the flow state proper also needs to keep participants on task. To this end, we measured best-try using the Effort/Importance subscale from the IMI. In all three experiments, best-try was high, and critically relatively well matched across high- and low-flow conditions. The best-try values observed were between 5.4 and 5.7 out of 7, which indicates relatively high effort/importance. These values are in line with those of Mihelj et al. (2012), who reported values around 5.9 from participants engaging in a rehabilitation video game, and 5.4 from participants in an aerobics class. Motivation to try their best was relatively high for both of these groups, as participants hoped to recover greater functions in the former group, and willingly participated in the aerobics class in the latter group. The physiological measures collected in E2 and E3 also suggest that physiological effort was matched in both flow conditions. Indeed, increased mental effort has been repeatedly associated with increased SCL, shallower and faster respiration (Backs & Seljos, 1994), increased heart rate, and a decrease in heart rate variability (Brouwer et al., 2015; Richter et al., 2016). The lack of physiological differences during the high- and low-flow conditions points to rather similar mental effort, in line with best-try, suggesting that participants were similarly engaged across flow conditions. A crucial aspect of the proposed procedure is that high best-try values and matched physiological states were observed from the high- and low-flow conditions in the face of decisive evidence for a difference in reported flow state with a larger effect size. Thus, the proposed procedure reliably induces within participants two different flow states while ensuring that participants remain on task across the two conditions.

This work also points toward differential views of what is termed “effort” in a variety of literature and how these constructs may relate. The first one, measured by our best-try measure, is the subject-related willingness to put effort toward the task in line with the intrinsic motivation scale it is taken from. Subjects’ willingness may be high or low for the same level of task difficulty. The second one is the subjective level of effort the participant estimates necessary to do the task well, as measured by the RSME. Unlike best-try, this measure pertains to the task-related influence on the participant’s state of mind. An easy task will lead to a low RSME rating, even if the subject is willing to put a great deal of effort toward it. The third and final one is the actual objective physiological states the body is in when performing a task as estimated by cardiac or skin conductance measures (Brouwer et al., 2015). In our study, participants reported similarly trying their best in both flow conditions (as measured by the Best-Try scale) but reported that play during the low-flow game took more effort than that during the high-flow one (as measured by the RSME). Interestingly, physiological measures such as HF-HRV did not differ across conditions, indicating similar objective effort produced toward the task, even in the face of greater subjective effort, as measured by RSME. It is worth noting that in the other studies that did not report differences in HF-HRV between their high-flow condition and low-flow difficult condition (de Sampaio Barros et al., 2018; Harmat et al., 2015), the challenge of the task in the high-flow condition was matched to the skills of the participant, while in the studies that did report such differences, the challenge of the task was simply set at arbitrary easy, medium, and hard levels in the “boredom,” “flow,” and “frustration” conditions, respectively. In such cases, the difficulty of the tasks may not be properly calibrated for each participant, with the frustration condition being too hard or the boredom condition too easy at least for some participants. Motivational intensity theory predicts that “objective effort” (namely the effort actually produced by the body) could become low in both the easy and the hard condition as participants may switch to off-task behavior. On the other hand, the subjective effort perceived to perform the task (measured for example with the RSME) will vary from low in the easy condition to high in the hard condition, with an intermediate value for the medium, high-flow condition. This is exactly the pattern of results that was found in Harris et al. (2017), whereby RSME followed a linear trend with task challenge, while HF-HRV showed a U-shaped pattern where it was low (higher effort) in the high-flow condition and higher (low effort) in the other two, easy and hard conditions.

By design, flow studies manipulate task difficulty through different skill-challenge levels—either a balanced level for flow or levels too easy/too hard for the baseline condition(s). The present work establishes that these baseline conditions can be designed to induce in the player the same motivation to try their best, ensuring matched conditions in terms of on-task behavior. Flow studies that do not address this issue are possibly confounded, as illustrated by a recent review on the neural correlates of boredom in which two out of the three studies were in fact studies primarily designed to study flow (Raffaelli et al., 2018). The procedure presented in this paper addresses this possible confound by allowing researchers to contrast conditions that differ in flow level, while maintaining participants similarly on task.

Finally, although our method was effective at inducing reliable flow differences across game conditions, our game titration method may be further improved. First, the screening *UT2004* game played before the main experiment is a one-on-one match instead of the five-player free-for-all game used in the main experiment. This means that participants who perform well on this mode may not perform as well on the high-flow and low-flow games that involve more opponents controlled by the computer. It may be advisable for the screening game to also be played in the same five-player free-for-all game play that the high- and low-flow conditions use. Second, while we looked for a relative balance between kills and getting killed via our adaptative procedure, it is possible that flow would be even higher had we allowed for greater KDR. This is certainly worth exploring in future studies. Third, the screening and evaluation games were played outside the scanner, in a sitting position with a standard computer setup, whereas in the scanner, participants played in supine position with the keyboard and mouse supported by a work surface at waist level. Although we had participants play a 5-minute adaptation game in the scanner to habituate to this unusual configuration, it may be preferable to administer the evaluation games in the very supine position the scanner demands. In addition, it is clear that starting with a high-flow game makes the low-flow game potentially less interesting; further studies of these order effects may be important as we aim at keeping participants trying their best, despite changes in flow level. Fourth, our measures of flow in the two conditions, while reliably different, covered only slightly more than one tenth of the whole range of the FSS (average difference of 0.5 for a scale ranging from 1 to 5). This restricted range of flow values may be due to the careful matching of best-try across conditions so as to unconfound flow from other off-task states, such as boredom or frustration. However, we are encouraged by the fact that other in-laboratory studies of flow reported an average of .48 between their “in-flow” and their “over-challenge” conditions (see supplementary materials SM-10), as well as the fact that the FSS showed excellent discriminating power between the high- and low-flow states as induced by our novel methodology. Finally, as is the case for all other flow-inducing activities, this method is suited neither for experts (KDR > 2 at the difficulty level 5/8 on UT) nor for novices (individuals unable to play video games).

In sum, this work presents a new method to induce both a state of high flow and one of low flow while keeping participants similarly on task and trying their best in both states. The proposed method is free and simple, relying entirely on the publicly available game *Unreal Tournament* 2004. It also has the advantage of being short, working in the absence of the game soundtrack, and being easily portable to the demanding conditions of brain imaging.

## Data Availability

The datasets generated during and/or analyzed during the current study, as well as supplementary materials, are available at https://osf.io/ukpdy/.
